# Low frequency ultrasound elicits broad cortical responses inhibited by ketamine in mice

**DOI:** 10.1038/s44172-024-00269-2

**Published:** 2024-08-27

**Authors:** Linli Shi, Christina Mastracchio, Ilyas Saytashev, Meijun Ye

**Affiliations:** Division of Biomedical Physics, Office of Science and Engineering Laboratories, Center for Devices and Radiological Health, Food and Drug Administration, Silver Spring, MD USA

**Keywords:** Neuronal physiology, Physiology

## Abstract

The neuromodulatory effects of >250 kHz ultrasound have been well-demonstrated, but the impact of lower-frequency ultrasound, which can transmit better through air and the skull, on the brain is unclear. This study investigates the biological impact of 40 kHz pulsed ultrasound on the brain using calcium imaging and electrophysiology in mice. Our findings reveal burst duration-dependent neural responses in somatosensory and auditory cortices, resembling responses to 12 kHz audible tone, in vivo. In vitro brain slice experiments show no neural responses to 300 kPa 40 kHz ultrasound, implying indirect network effects. Ketamine fully blocks neural responses to ultrasound in both cortices but only partially affects 12 kHz audible tone responses in the somatosensory cortex and has no impact on auditory cortex 12 kHz responses. This suggests that low-frequency ultrasound’s cortical effects rely heavily on NMDA receptors and may involve mechanisms beyond indirect auditory cortex activation. This research uncovers potential low-frequency ultrasound effects and mechanisms in the brain, offering a path for future neuromodulation.

## Abstract (web summary)

Dr Ye and colleagues investigate the biological impact low-frequency ultrasound pulses can have on the cortex of mice. They observe pulse duration dependent neural responses and find that ketamine can block parameter-dependent brain responses at certain frequencies.

## Introduction

Ultrasound is gaining recognition as a non-invasive neuromodulation tool by virtue of its unique advantages, including high spatial resolution and deep tissue penetration. Currently, the most widely used ultrasound frequencies for transcranial neuromodulation range from 250 to 500 kHz^1,2^. To ensure sufficient energy can be transferred to the neural tissue to induce a neuromodulation effect, direct coupling of the ultrasound transducer and the target will be required. Lower frequencies of ultrasound, such as 20 kHz or 40 kHz, offer the benefit of air transmission and reduced skull attenuation, potentially facilitating more efficient coupling with lower thermal effect. However, due to the low spatial resolution and potential cavitation risk, there has been limited enthusiasm for employing low-frequency ultrasound for neuromodulation, accompanied by a paucity of research on its impact on the central nervous system. Results on this topic remain inconclusive.

Studies have shown varied effects of low-frequency ultrasound on both animals and humans. For instance, Schneider et al.^3^ found 20 kHz continuous-wave (CW) ultrasound had a parameter-dependent damaging effect in the rat head, with ≥0.5 W/cm^2^ (equivalent to 1.4 kPa) leading to magnetic resonance tomographic signal changes with negligible temperature increase of 0.2 °C. A human study using a 20 kHz CW ultrasound with a pressure level of 84 dB (equivalent to 0.3 Pa) showed no symptoms^4^. Di Battista et al.^5^ reported that 120 dB (equivalent to 20 Pa) 40 kHz ultrasound had no detectable effect on human cognitive task performance. In another study, 500-ms bursts of 40 kHz ultrasound at up to 120 dB did not affect the behavioral or electrophysiological measures of auditory functions in humans^6^. However, two other studies suggested that in a subpopulation very high-frequency sound and ultrasound exposures can disrupt sustained attention and increase the anxiety at pressure levels between 82 and 92 dB^7,8^. These discrepancies emphasize the need for further investigation into the biological effects of low-frequency ultrasound to ensure its safe use and explore potential therapeutic applications.

Multiple hypotheses have been postulated for the underlying biophysical mechanisms of higher frequency ultrasound (e.g., >200 kHz) neural effects, including local temperature increase^9^, transient sonoporation^10,11^, intramembrane cavitation^12,13^, mechanosensitive ion channel activation on glia or neurons^14–17^, and auditory pathway activation^18,19^. Recent research using low-intensity pulsed ultrasound has demonstrated neuromodulation effects with minimal temperature increases (< 0.1 °C), challenging the thermal theory^20–22^. The involvement of mechanosensitive ion channels, including TREK-1, TREK-2, TRAAK, Nav1.5^14^, TRP-4^23^, TRPP2, TRPC1, TRPA1, and Piezo1, has been well established^16–19^ The auditory pathway has gained prominence in explaining transcranial ultrasound neuromodulation^18,19^.

Another critical factor in ultrasound-based experiments is the choice and depth of anesthesia, which can impact outcomes. For instance, ketamine has been shown to inhibit ultrasound-induced motor responses and Ca^2+^ transients^24^, suggesting a network effect.

While the effects of low-frequency ultrasound’s neural effects are less comprehensively understood compared to high-frequency ultrasound, given the capability of higher transcranial transmission efficiency and lower threshold for cavitation, it is possible that they can produce cavitation, sonoporation, activation of mechanosensitive receptors, and activation of auditory pathway as well. Thus, it is plausible to hypothesize that similar neural effect can be evoked by low-frequency ultrasound.

This study aimed to shed light on the biological impact of low-frequency ultrasound on the mouse cortex with implementation of Ca^2+^ imaging and electrophysiological recording in vivo and in vitro. Neuronal responses to different dosages of pulsed 40 kHz ultrasound were investigated in the somatosensory (SC) and auditory (AC) cortices and compared with those to 12 kHz audible tone sound exposures. Furthermore, the influence of isoflurane and ketamine on these responses was studied. These results suggest that low-frequency ultrasound can possibly modulate brain activities through network effect. This study implies another avenue for future research in neuromodulation, particularly with the development of ultrasound multi-element arrays^25^, which embrace the capability of focusing low-frequency ultrasound to a small area.

## Results

### Ultrasound transducer characterization

**Fig. 1:**
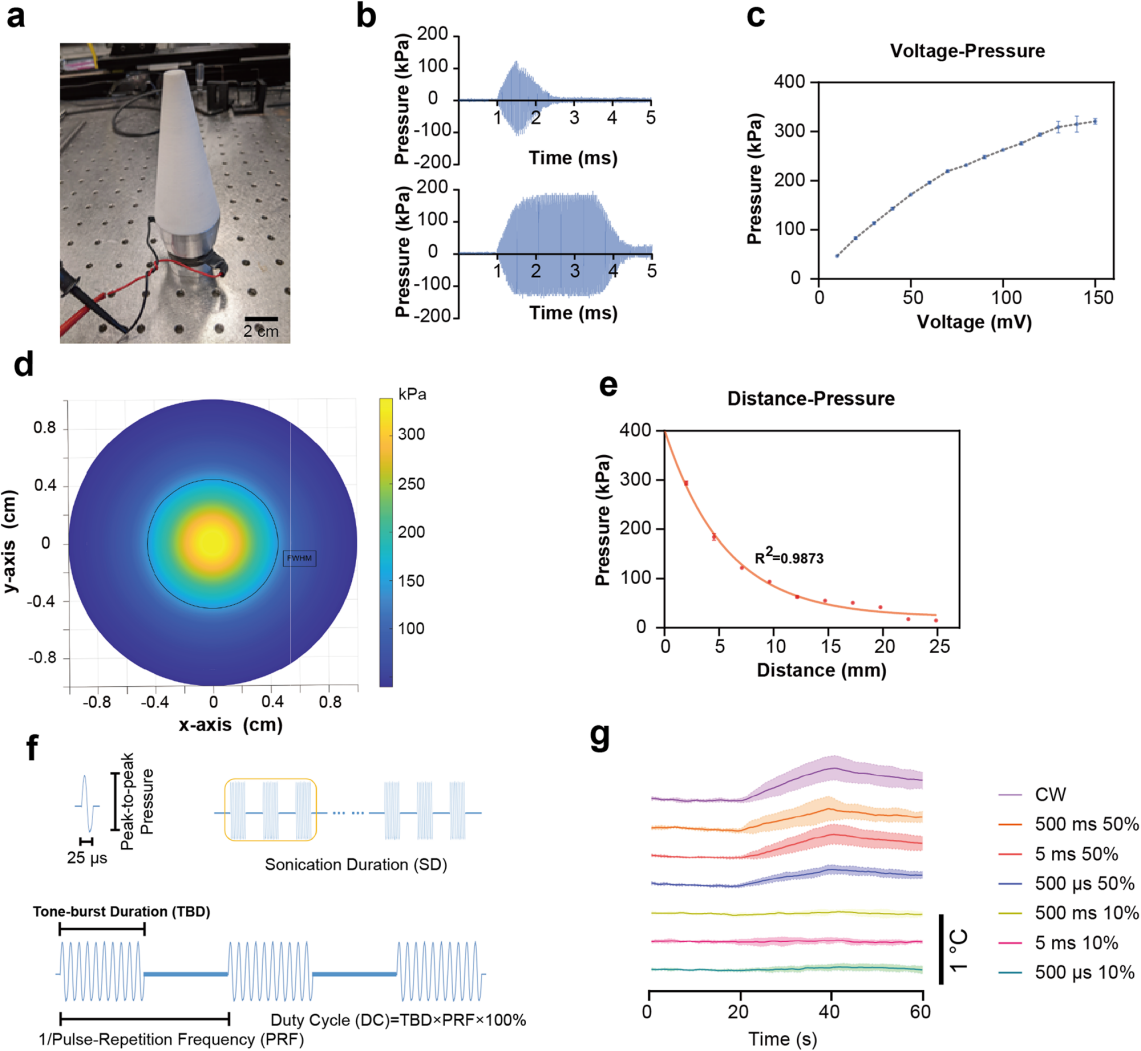
Characterization of the 40 kHz ultrasound transducer. **a** Image of the 40 kHz transducer with a cone-shaped waveguide. **b** Representative waveforms for bursts of 0.5 ms and 2.5 ms durations, showing that the steady peak-to-peak (P-P) pressure is reached at about 0.7 ms after ultrasound onset. **c** Relationship between input voltage and output P-P pressure, indicating a maximum system-generated pressure of about 300 kPa. **d** Mapping of the acoustic pressure in the radial direction suggesting a full width half maximum (FWHM) of about 8 mm. **e** Pressure decay in the axial direction measured at 8.4 mm. **f** Illustration of the ultrasound tone burst. Single sine wave with 25 µs was delivered continuously with specific tone-burst-duration (TBD). The duty cycle (DC) is defined as the TBD divided by the 1/pulse repetition frequency (PRF)*100%. The total sonication time is defined as the sonication duration (SD). **g** Intracranial temperature rises during ultrasound exposure (data expressed as mean ± SEM, *n* = 3 for the pressure and temperature measurement).

A 40 kHz transducer with a custom-made coupling cone was used in the present work to generate pulsed ultrasound. The temporal and pressure profile of the 40 kHz transducer (Fig. 1a) was characterized in a degassed water tank. The steady peak-to-peak pressure reached approximately 0.7 ms after the onset of the burst. Representative waveforms of 0.5 ms and 2.5 ms duration bursts are shown in Fig. 1b. The peak-to-peak pressure can be fine-tuned by varying the driving voltage (Fig. 1c). As mapped out by hydrophone measurements in the radial direction, the full width half maximum (FWHM) of the acoustic field is around 8 mm (Fig. 1d). In the axial direction, the pressure decays to 1/e at a distance around 8.4 mm (Fig. 1e). Figure 1f illustrates the terms used to describe the parameter space of the ultrasound throughout the paper. The pressure described in the present work refers to Peak-to-peak pressure unless otherwise noted.

To determine the attenuation rate of the mouse skull, we measured the pressure within three mouse cranial vaults in water. In all, 60–90% transmission rate through the skull at the unreflected entry pulse was measured, while the peak reflected pressure was 40–70% of the free field (Supplementary Fig. 1). The 60% transmission occurred at a time of 2 × 10^−4^ s. And higher transmission was observed at the beginning and end of the pulse.

For a better understanding of the acoustic field, a simulation toolbox, k-Wave^26^, was used to generate simulations of the acoustic propagation inside the skull (see Methods in Supplementary Information). The transducer cone is placed close to the skull to match the in vivo conditions (Supplementary Fig. 2). As shown in the coronal view and horizontal view, the acoustic signal did not present constructive and destructive interference, with a decay profile similar to the experimental result in Fig. 1e, which is found reasonable due to the low absorption coefficient and long wavelength of ~37.5 mm for 40 kHz. Furthermore, according to the experimental measurement of intracranial pressure inside the mouse cadaver (Supplementary Fig. 1), the transmission efficiency of 60–90% matches the simulation result. Thus, both experiment and simulation present a smooth distribution of the acoustic signal in the mouse head gradually decaying along the path.

As thermal effect is one of postulated ultrasound neuromodulation mechanisms, we measured the temperature rise during ultrasound exposure using a miniaturized thermocouple (DI-245, DataQ Instruments, USA) embedded within the cranium of mouse cadavers. Figure 1g illustrates the temperature elevation, showing a maximum increase of less than 1 °C under the highest dosage (20 s of 300 kPa) of continuous-wave ultrasound. Considering the absence of perfusion in the cadavers, it is reasonable to anticipate that the actual temperature rise would be even lower.

### 40 kHz pulsed ultrasound increases neuronal activities in the somatosensory cortex in vivo in a tone-burst duration-dependent manner

**Fig. 2:**
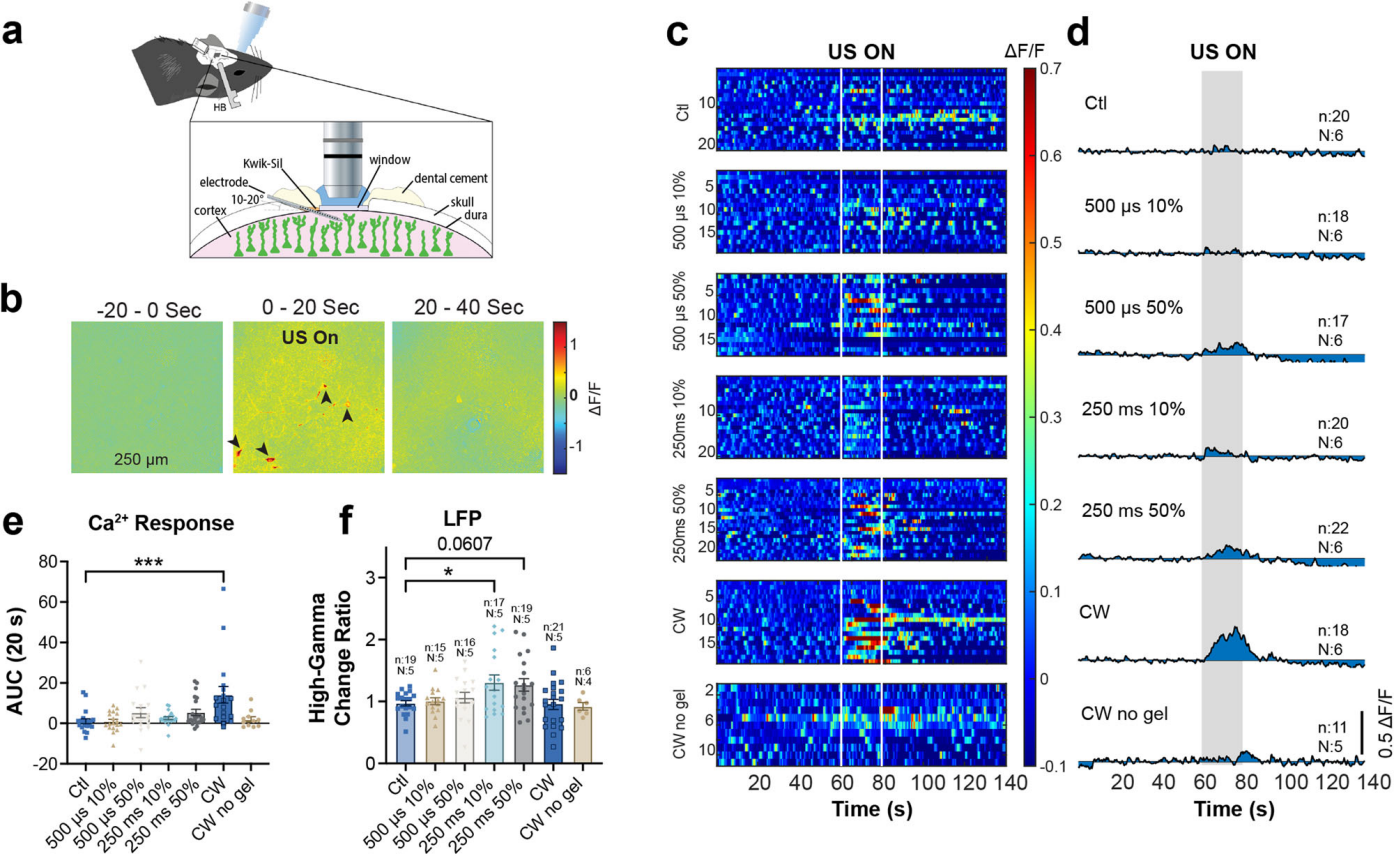
40 kHz ultrasound exposure increased neuronal activity at the somatosensory (SC) cortex in vivo in a parameter-dependent manner. **a** Illustration of the experimental setup, the window surgery, electrode implantation and the placement of ultrasound transducer. **b** An example full field Ca^2+^ signal (ΔF/F) before, during and after 50% duty cycle 250 ms tone burst duration (TBD) ultrasound exposure, averaged across 20-s duration. Note that only a subset of cells (arrowhead indicated) responded. **c** Heatmaps showing Ca^2+^ signals of the average of top ten responsive cells in each trial in ΔF/F. Each row is a trial. **d** Traces of the mean Ca^2+^ signal for each parameter set in the heatmap, which demonstrates a clear parameter-dependent relationship between ultrasound parameters and neuronal responses. (*n* trial number, *N* animal number). **e** Statistical summary of area under the curve (AUC) of Ca^2+^ signals during ultrasound exposure for each parameter set. Kruskal–Wallis test revealed significant group difference among parameters (*P* < 0.005). Dunn’s multiple comparison test showed non-significantly larger AUC for both 500 µs and 250 ms 50% duty cycle, and significantly larger AUC for continuous wave (CW) group (*P* < 0.001). **f** Local field potential (LFP) changes at high-gamma (64–100 Hz) frequency band during ultrasound exposure. One-way ANOVA indicated significant group difference among parameters (*P* < 0.05). Dunnett’s multiple comparison showed significant increase in high-gamma oscillations for 10% 250 ms group and non-significant increase for 50% 250 ms exposure (data in (**e**, **f**) are expressed as mean ± SEM, and each dot represents each trial. *n* trial number, *N* animal number).

To investigate neuronal responses to 40 kHz pulsed ultrasound exposures, a glass window was implanted on the somatosensory cortex (SC) of adult transgenic mice expressing Ca^2+^ indicators (Thy1-GCaMP6s) in excitatory neurons. A single-shank, 16-channel, Michigan-style microelectrode array (A1X16-3mm-100-703-CM16LP, NeuroNexus, Ann Arbor, MI) was implanted at an angle of 10–20° relative to the brain surface into the layer 2/3 SC for simultaneous electrophysiological recording. Ultrasound was delivered through a transducer placed on the ipsilateral side of the imaging window under isoflurane anesthesia at the highest energy level with approximately 300 kPa of pressure (Fig. 2a). For each ultrasound exposure trial, we acquired 60 s of baseline, 20 s of ultrasound exposure, and 60 s of post-exposure Ca^2+^ signals. The dosages are summarized in Table 1.

**Table 1:** Ultrasound parameters tested in vivo

	500 µs 10%	5 ms 10%	250 ms 10%	500 µs 50%	5 ms 50%	250 ms 50%	CW
Carrying frequency	40 kHz	40 kHz	40 kHz	40 kHz	40 kHz	40 kHz	40 kHz
Tone burst duration	500 µs	5 ms	250 ms	500 µs	5 ms	250 ms	Continuous wave (CW)
Pulse repetition frequency	200 Hz	20 Hz	0.4 Hz	1000 Hz	100 Hz	2 Hz	N/A
Duty cycle	10%	10%	10%	50%	50%	50%	100%
Peak-to-peak pressure (in water)	235 kPa	300 kPa	300 kPa	235 kPa	300 kPa	300 kPa	300 kPa
Mechanical Index (MI)	0.54	0.65	0.65	0.54	0.65	0.65	0.65

Full field intensity analysis demonstrated an apparent increase in the Ca^2+^ signal during higher parameters, specifically with a 250 ms ultrasound tone-burst-duration (TBD). Some cells exhibit higher responsiveness than others, as shown in Fig. 2b. Therefore, we conducted individual cell response analysis. The ten most responsive cells were determined by the MATLAB code based on the Ca^2+^ event change ratio from each trial. The heatmaps in Fig. 2c display the average responses of the top ten responsive cells in each trial under different exposure dosages, revealing a clear parameter-response relationship.

To quantify the Ca^2+^ signal change, we calculated the ΔF/F_0_ for the ten most responsive cells in each trial, and then determined the area under the curve (AUC) during the 20 s ultrasound exposure, as indicated by the shaded area in Fig. 2d. In the control group, no ultrasound was applied, and Ca^2+^ images were collected for the same duration, 140 s. Kruskal–Wallis test revealed a significant group difference among different parameters (trial and animal numbers are shown in Fig. 2d). Dunn’s multiple comparison test showed that 50% duty cycle groups, including both 500 µs and 250 ms tone burst duration (TBD) groups, had a non-significantly larger AUC during exposure compared to the control group (Fig. 2e, *P* = 0.1011 for the 50% duty cycle of 250 ms TBD, *P* = 0.3758 for 10% 250 ms, and *P* = 0.2989 for 50% 500 µs). The AUC of the continuous wave (CW) group was significantly larger than that of the control group (Fig. 2e, *P* < 0.001).

The Ca^2+^ imaging analyses indicate an increased neuronal activity during ultrasound exposure. However, Ca^2+^ imaging can only visualize limited fields of the layer 2/3 cortex, and only excitatory cells were labeled. Few preliminary studies suggest that CW affects neuronal activities differently from pulsed ultrasound^27,28^ and that different neuron populations can have specific responsive ultrasound parameter sets^29,30^. To gain a more comprehensive understanding, we further analyzed electrophysiological signals collected via implanted electrodes that spanned the entire cortical layers and are not cell type specific.

Local field potential (LFP) analyses revealed that 250 ms pulsed ultrasound increased the full spectrum of LFP from 1 to 100 Hz, regardless of the duty cycle, but not the 500 µs pulses (Supplementary Fig. 3). To further quantify the data, we averaged the change in power spectral density (PSD) for different frequency bands, including delta (1–4 Hz), theta (4–8 Hz), alpha (8–13 Hz), beta (13–30 Hz), low-gamma (30–56 Hz), and high-gamma (64–100 Hz) (Supplementary Fig. 3). To exclude the possible effect of 60 Hz noise on the power estimation, signals between 56 and 64 Hz were removed from the band quantification. We calculated PSD change ratios by dividing the average PSD during exposure by the average PSD before exposure at various frequency bands. Significant group difference in the high-gamma (64–100 Hz) band was revealed by one-way ANOVA. Dunnett’s multiple comparison test showed a significant increase in high-gamma oscillations for the 10% 250 ms group (*P* < 0.05) and a non-significant increase for the 50% 250 ms exposure (*P* = 0.0607) (Fig. 2f and Supplementary Fig. 3). However, unlike the observed increase in Ca^2+^ signals, CW did not induce a significant change in LFP. This can be attributed to the cell type specificity of the ultrasound response^27–30^. In addition, variability in the correlation between individual cell activity and LFP has been documented^31^.

As the peak pressure of ultrasound would not reach a steady status until ~0.7 ms after the onset, as shown in Fig. 1b, for shorter TBD (i.e., 500 µs), the maximal pressure can be lower than that of the longer TBD (i.e., 250 ms) (Table 1). To ensure that the different response magnitudes we observed between 500 µs and 250 ms TBDs were not solely due to pressure difference, we included a 5 ms exposure in a separate group of animals. We choose not to combine results from these two groups of animals to mitigate animal and experimental variability, as evident in Fig. 2c, e, f. Consistent with the observations described above, 250 ms ultrasound induced the highest responses in both Ca^2+^ and LFP analyses (Supplementary Fig. 4).

These data suggest that 40 kHz ultrasound alters both individual and population neuronal activity during the exposure in the SC. The degree of the responses is highly correlated with the TBD of the ultrasound, and the duty cycle may also influence the response.

Since 40 kHz falls within the hearing range for mice, we attempted to exclude the possibility that the observed effects were a result of indirect cortico-cortical excitation from the AC in response to sound. Thus, we removed the ultrasound gel between the mouse’s head and the transducer and acquired Ca^2+^ images and electrophysiological recordings during CW exposure, which induced the strongest Ca^2+^ responses when using gel coupling. In the absence of acoustic coupling, no Ca^2+^ activity or high-gamma increase was detected, as depicted in Fig. 2c–f and Supplementary Fig. 3 (*P* > 0.9999 for Ca^2+^ analysis and *P* > 0.9995 for LFP). This result implies that neuronal responses observed in the SC were not a result of air-transduced hearing response to 40 kHz ultrasound.

### Comparable neuronal responses in the auditory cortex to 40 kHz pulsed ultrasound and 12 kHz sound

While our results with the decoupling of the transducer from the head suggest that responses evoked by 40 kHz pulsed ultrasound in the SC were not due to air-transduced hearing mechanism as how sound is heard, it is known that ultrasound can possibly activate the auditory pathway through bone transduction. This has been elegantly demonstrated by Guo et al. and Sato et al.^18,19^. Therefore, we tested whether 40 kHz pulsed ultrasound could evoke responses in the AC and compared them with responses to 12 kHz audible sound stimuli. In this experiment, we only placed a glass window at the AC without implanting an electrode.

**Fig. 3:**
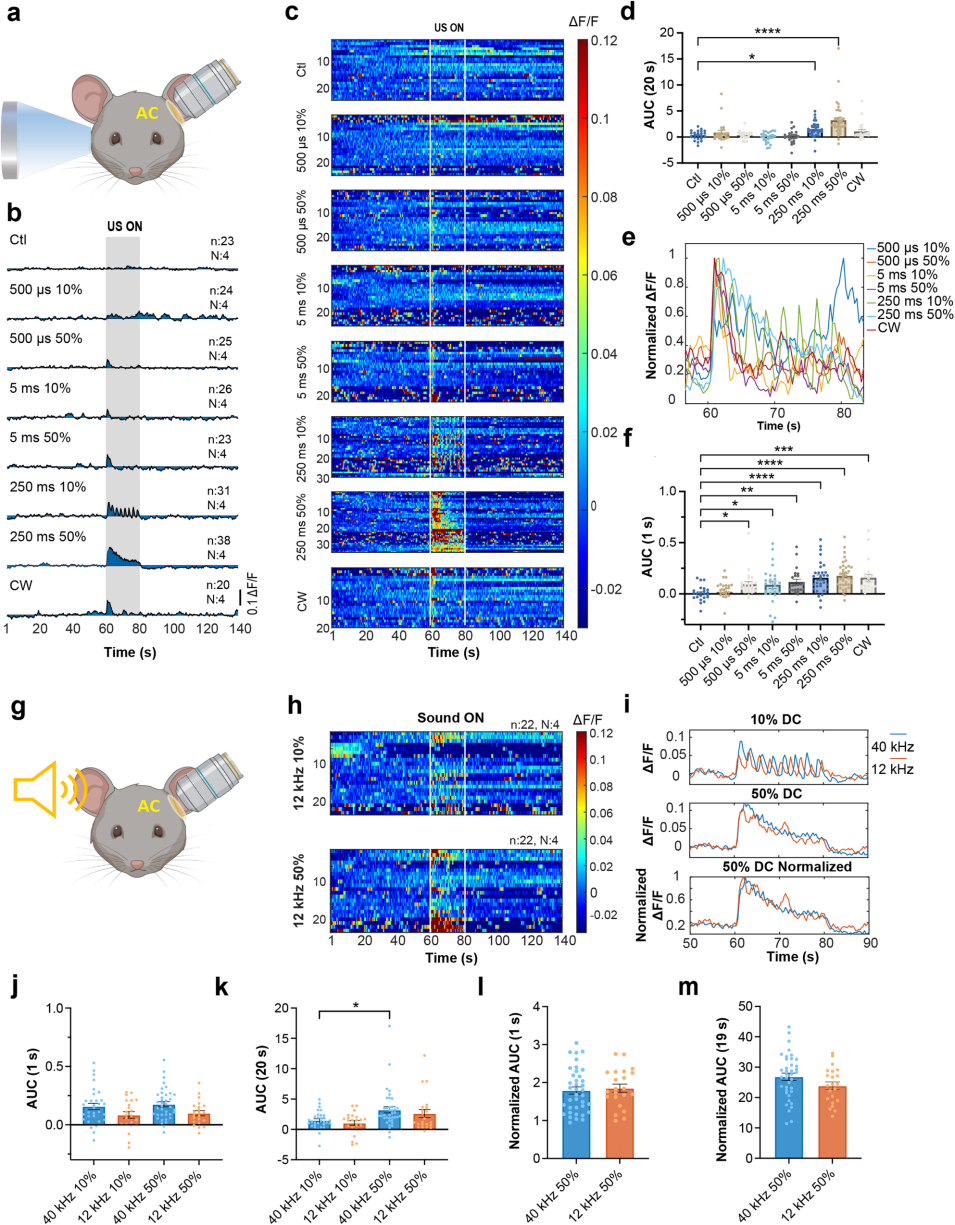
Comparable neuronal responses in the auditory cortex to 40 kHz pulsed ultrasound and 12 kHz sound. **a** Illustration of experimental paradigm for imaging Ca^2+^ responses to 40 kHz ultrasound exposures. **b** Traces of the mean Ca^2+^ responses to each ultrasound paradigm. **c** Heatmaps showing Ca^2+^ signals of the average of top ten responsive cells in each trial, presented in ΔF/F. Each row represents one trial. **d** Statistical summary of area under the curve (AUC) of Ca^2+^ signals during 20-s ultrasound exposure for each exposure paradigm. Significantly larger AUC for 10% and 50% of 250 ms TBD exposures compared to control were revealed. All other exposures showed no significant difference from the control (*P* > 0.9999). **e** The average of the normalized Ca^2+^ traces to the peak responses for different ultrasound exposure parameters. **f** Statistical summary of the AUC of the 1st-second Ca^2+^ responses. All exposures, except for 10% 500 µs TBD, had significantly larger AUC compared to the control, indicative of a transient response to 40 kHz ultrasound. **g** Illustration of experimental paradigm for imaging Ca^2+^ responses to 12 kHz ultrasound exposures. **h** Heatmaps of Ca^2+^ signals of the top ten responsive cells in each trial in response to 12 kHz 75 dB sound exposures. **i** Comparison of Ca^2+^ responses to 40 kHz ultrasound versus 12 kHz sound exposures. Top: 10% duty cycle (DC), middle: 50% DC, bottom: normalized 50% DC. **j** AUC of the 1st-second Ca^2+^ responses to 250 ms TBD ultrasound and sound at 10% and 50% DC, respectively. AUCs of the 40 kHz group were larger than those of 12 kHz group (*P* = 0.1744 for 10% DC and *P* < 0.05 for 50% DC). No differences were detected between 10% and 50% of ultrasound or between 10% and 50% of sound (*P* > 0.5). **k** AUC of 20-s Ca^2+^ responses to ultrasound and sound at 10% and 50% DC, respectively. No significant difference was observed between ultrasound and sound with the same duty cycle. However, the 50% group show overall higher responses than the 10% group (*P* < 0.01 for ultrasound and *P* = 0.2887 for sound). **l**, **m** AUC of 1st and 2–20 s normalized Ca^2+^ signals for 50% ultrasound and sound, respectively (statistics: Kruskal–Wallis and Dunn’s multiple comparison tests in (**d**, **f**, **j**, **k**). Mann–Whitney test in (**l**, **m**). *P < 0.05, **P < 0.01, ***P < 0.001, ****P < 0.0001. Data in (**d**, **f**, **j**–**m**) are expressed as mean ± SEM, and each dot represents one trial. *n* trial number, *N* animal number).

As anticipated, contralateral ultrasound exposure through the coupling gel induced apparent responses at the AC. Quantitative analysis revealed a significant increase in the AUC for 10% and 50% duty cycle 250 ms TBD during 20-s exposures (Fig. 3a–d). Though AUCs for other parameters did not significantly differ from the control, Ca^2+^ traces (Fig. 3b) and heatmaps (Fig. 3c) displayed evident but more transient responses. To better understand the dynamics of responses to different ultrasound parameters, we normalized the ΔF/F to the peak response (Fig. 3e). Responses to all parameters exhibited a similar rising pattern and peaked at around 1 s after the onset of exposure. However, it needs to be noted that our frame rate was three frames per second, which may prevent the detection of more subtle dynamic differences. Hence, we compared the AUC of the first-second response among groups. Except for 10% duty cycle 500 µs TBD, all exposures elicited Ca^2+^ responses significantly higher than the control (Fig. 3f). No significant difference was observed between 10% and 50% duty cycles of the same TBDs in 1-s AUC analysis. However, the 250 ms TBD generated significantly larger responses compared to the same duty cycle of 500 µs (*P* < 0.01 for 10% duty cycle, *P* < 0.05 for 50% duty cycle) in the 1-s AUC analysis, as well as larger responses than both 500 µs and 5 ms TBDs in the 20-s analysis (*P* < 0.001, Kruskal–Wallis and Dunn’s multiple comparison). These analyses suggest Ca^2+^ responses in the AC are more dependent on the TBD rather than the duty cycle.

In addition, to investigate whether the responses to 40 kHz ultrasound were similar to those elicited by audible sound, we exposed the same animals to 10% and 50% duty cycle 12 kHz sound at 75 dB, which evoked responses comparable to those seen with 250 ms TBD ultrasound (Fig. 3g–i). AUC quantification revealed no significant difference between sound and ultrasound with the same duty cycle within the first second or throughout a 20-s exposure (Fig. 3j, k) (*P* = 0.7910 for 10% duty cycle, *P* = 0.1676 for 50% in the first-second analysis. In the 20-s analysis, *P* > 0.9999 for 10%, *P* = 0.4903 for 50%, Kruskal–Wallis and Dunn’s multiple comparison). However, 50% duty cycle ultrasound demonstrated significantly larger responses than 10% in the 20-s analysis (Fig. 3k) (*P* < 0.05, Kruskal–Wallis and Dunn’s multiple comparison). To compare the dynamics, we normalized responses to 50% sound and ultrasound, and calculated the AUC for the normalized data in the first second and between 2 and 20 s of exposure. No significant difference was observed between the two groups (Fig. 3i, l, m), suggesting similar dynamics.

As removing the direct coupling between the transducer and the mouse head eliminated the response in the somatosensory cortex, we further investigated whether this would also affect the responses in the AC. Unlike in the SC, responses to 50% duty cycle 250 ms TBD, which generated largest responses in the direct coupling configuration, were significantly reduced in the AC, but not completely abolished (Supplementary Fig. 5). This result was somewhat expected, as 40 kHz is still within the mouse’s hearing range^32^.

However, this raises a question: Does the elimination of responses in the SC when the transducer is decoupled stem from a remarkably reduced auditory response, or is it due to the SC being activated through a different pathway rather than indirect activation via the AC? To explore this, we compared the Ca^2+^ responses to 40 kHz ultrasound between the SC and AC to understand their relationship. We identified a significantly lower magnitude of response in the SC (Supplementary Fig. 6a, b), manifested as significantly lower AUC in both the first second and the 2–20 s durations (Supplementary Fig. 6b–d) (Mann–Whitney test). Normalization of the Ca^2+^ responses to the peak amplitude revealed a similar onset between responses in the SC and AC, but a slower decay in the AC (Supplementary Fig. 6b, bottom panel). This was further verified by calculating the AUC of the normalized signals, in which no significant difference in the first second was detected, while the AC had a significantly larger AUC of the later 19-s normalized signals than the SC (Supplementary Fig. 6e, f) (Mann–Whitney test).

### Lack of direct neuronal responses to 40 kHz ultrasound in brain slices

While the similar dynamics but lower amplitude of Ca^2+^ responses in the SC suggest that the reduced AC response in the air-transduction condition could lead to an undetectable response in the SC, this does not rule out other potential mechanisms, such as direct activation of neurons. Therefore, we conducted an assessment to determine whether 40 kHz ultrasound could directly activate SC neurons in brain slices obtained from thy1-GCaMP6s mice.

**Fig. 4:**
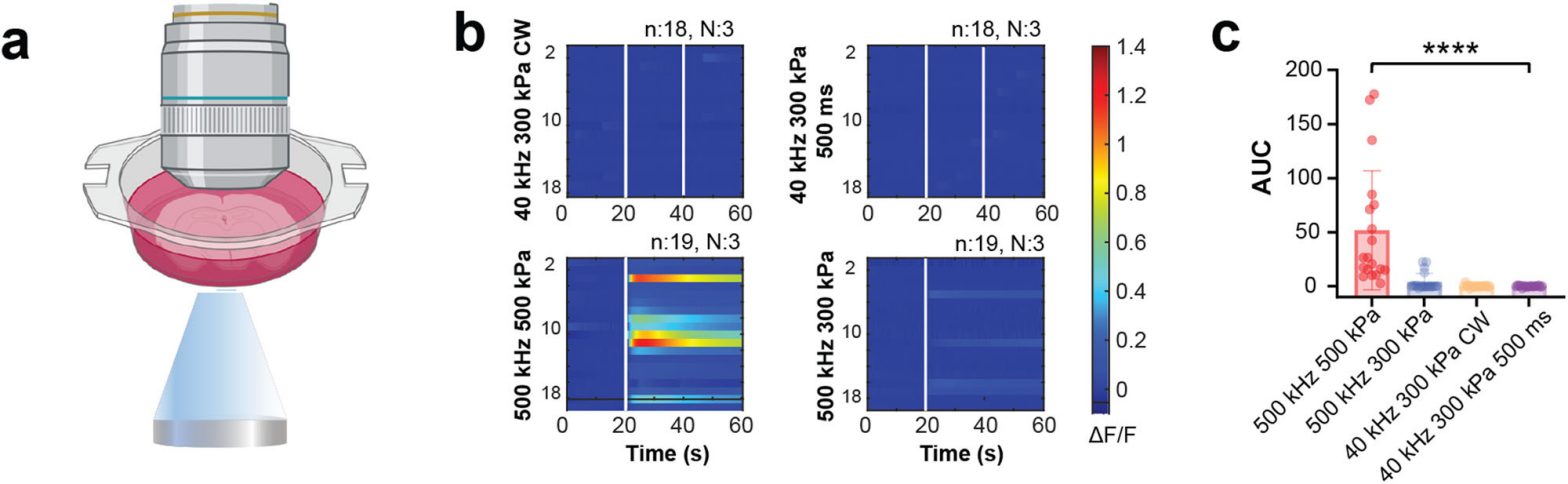
Lack of direct neuronal responses to 40 kHz ultrasound in brain slices. **a** Depiction of the In vitro experimental platform where ultrasound was delivered from the bottom of the slice perfusion chamber. **b** Heatmaps of Ca^2+^ responses to ultrasound exposure. For the 500 kHz condition, with only 1-s exposure, a single indication line is shown. **c** Quantitative comparison of the area under the curve (AUC) of Ca^2+^ responses for 20-s duration after the onset of ultrasound (data expressed as mean ± SEM, statistics: Kruskal–Wallis and Dunn’s multiple comparison tests, ****P < 0.0001).

We started with parameters employed in in vivo experiments. However, none of them elicited Ca^2+^ responses in brain slices. Given the observed correlation between Ca^2+^ response and TBD, we further increased the TBD to 50% duty cycle 500 ms and even CW. Yet, these parameters failed to induce observable neural activity, in contrast to our in vivo findings (Fig. 4). To rule out the possibility that the inability to induce neuronal responses was due to limitations of the in vitro platform, we introduced a 500 kHz ultrasound group—a frequency range known to successfully produce neuromodulation effects in vitro^16,17,33^. At the same pressure of 300 kPa and with a total exposure duration of 500 ms (100 ms burst duration, 50% DC, 5 bursts), we detected Ca^2+^ signals, albeit with a low success rate and low amplitude. The success rate and response amplitude increased when the pressure was raised to 500 kPa (Fig. 4b, c). Unfortunately, 300 kPa is the upper limit of our 40 kHz transducer. Consequently, we could not assess whether higher pressures at 40 kHz could possibly directly activate neurons. Nonetheless, these findings imply that the neural effects observed in vivo at 40 kHz were not a result of direct activation of neurons in the cortex.

### Similar responses to 40 kHz ultrasound and 12 kHz sound in the somatosensory cortex

Since 12 kHz sound and 40 kHz ultrasound induced similar responses in the AC, we hypothesized that they might also elicit similar responses in the SC if the response in the SC is transmitted from the AC. To investigate this, we compared the Ca^2+^ and LFP responses to sound and ultrasound in the SC. Only animals exposed to both sound and ultrasound are included in this analysis.

Both 10% and 50% duty cycle 12 kHz sound at 75 dB evoked noticeable Ca^2+^ responses and an overall increase in LFP broadband power in the SC (Supplementary Figs. 7a–c and 10a, b). Analysis of the amplitude and dynamics of Ca^2+^ responses indicated that the SC exhibited a comparable amplitude of responses to sound and ultrasound (Supplementary Fig. 7d, e, top panels). However, responses to sound appeared to have a faster onset and decay, as indicated by a non-significant higher 1st-second normalized AUC (*P* = 0.0554) and a significantly lower later 19-s (2–20 s) normalized AUC for the 12 kHz sound group (Supplementary Fig. 7d, e, bottom panels) (Mann–Whitney test). Additionally, the LFP changes induced by sound and ultrasound showed no significant difference in any frequency band (Supplementary Fig. 7f) (unpaired *t* test).

As responses to ultrasound in the SC appeared to have a lower magnitude and faster decay compared to the AC, we also compared responses to sound in the two regions. They exhibited a similar onset, but responses in the SC showed a faster decay (Supplementary Fig. 8). This was evident by a non-significant difference in the first-second AUC between the AC and SC, while a significantly larger AUC was observed in the AC compared to the SC (Supplementary Fig. 8b).

In summary, sound and ultrasound elicit similar responses in the SC, with sound showing a faster onset and decay. Compared with responses in AC, the sound induced responses in the SC decay faster, suggesting distinct processing dynamics in these two regions.

### Ketamine blocks neuronal responses to ultrasound and sound in the somatosensory cortex

Both Ca^2+^ imaging and LFP data demonstrate that 40 kHz ultrasound and 12 kHz sound can induce similar neuronal responses in the SC and AC under isoflurane anesthesia, which affects a broad range of neural pathways with primary involvement of GABA_A receptors. Some studies on ultrasound neuromodulation have suggested a role for the NMDA receptor^17,24^. To explore how the NMDA pathway affects these observed responses, we transitioned from isoflurane anesthesia to a ketamine/xylazine cocktail, which served as a NMDA receptor antagonist, and re-evaluated the effects of ultrasound and sound on SC neurons.

**Fig. 5:**
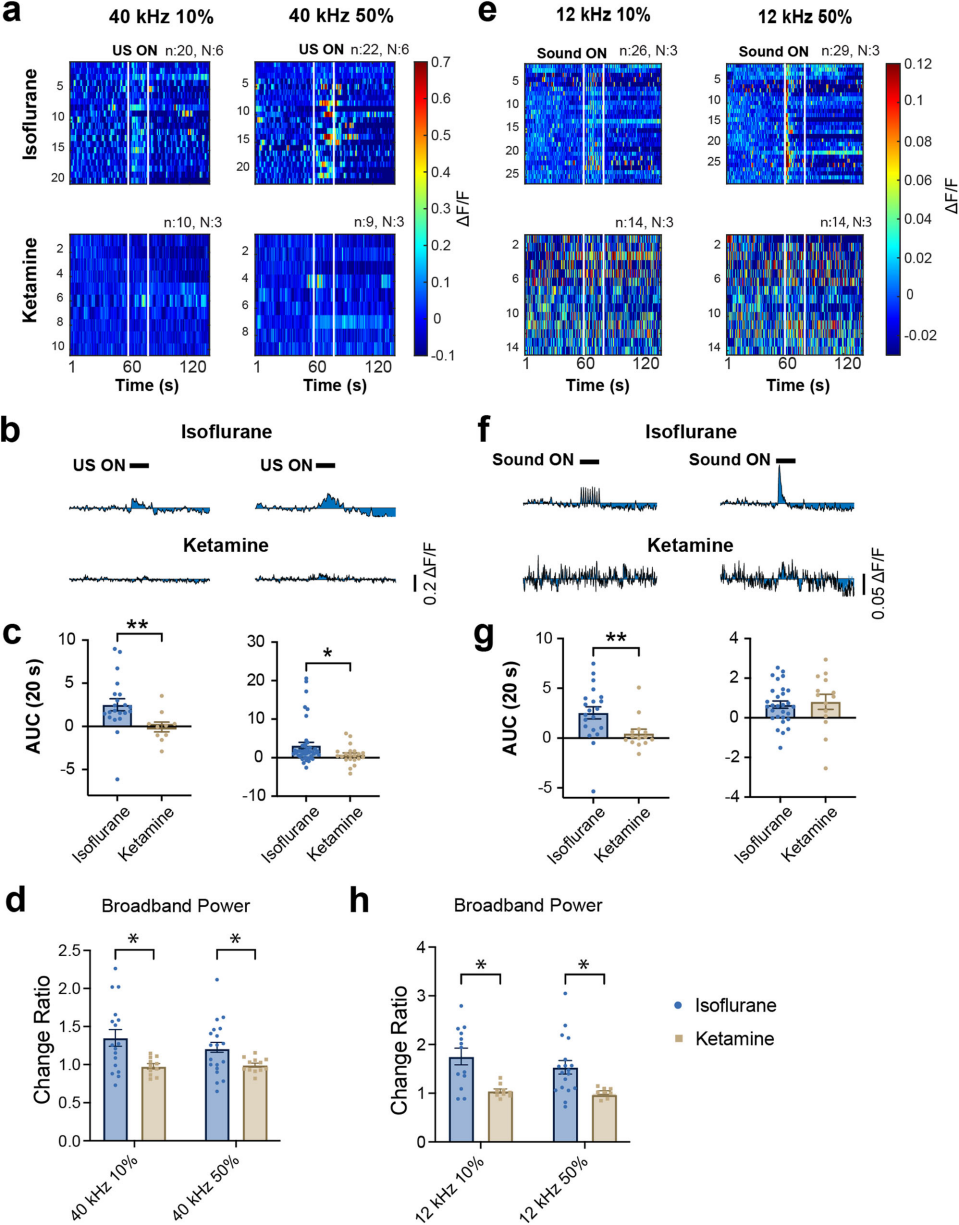
Ketamine blocked neuronal responses to ultrasound and sound in the somatosensory cortex. **a** Heatmaps showing Ca^2+^ responses to 10% and 50% 40 kHz ultrasound under isoflurane and ketamine anesthesia. **b** Average Ca^2+^ traces in response to 10% and 50% 40 kHz ultrasound under isoflurane and ketamine. Note that no apparent increase in Ca^2+^ signals under ketamine. **c** Area under the curve (AUC) during the 20-s ultrasound exposure comparison between isoflurane and ketamine. Significantly lower AUCs in ketamine were observed. **d** Comparison of LFP change induced by ultrasound between isoflurane and ketamine anesthesia. Significant differences were identified for broadband LFP (40 kHz 10%: *n* = 17, *N* = 5 in isoflurane, *n* = 11, *N* = 5 in ketamine; 40 kHz 50%: *n* = 19, *N* = 5 in isoflurane, *n* = 12, *N* = 5 in ketamine). **e** Heatmaps showing Ca^2+^ responses to 10% and 50% duty cycle 12 kHz sound under isoflurane and ketamine anesthesia. **f** Average Ca^2+^ traces in response to 10% and 50% sound under isoflurane and ketamine. Note that no apparent increase in Ca^2+^ signal for 10% duty cycle exposure and a slight increase in Ca^2+^ signal for 50% duty cycle under ketamine. **g** AUC during 20-s sound exposure comparison between isoflurane and ketamine. Significantly lower AUC for 10% duty cycle sound under ketamine was detected. However, no significant difference in response to 50% sound was revealed between isoflurane and ketamine. **h** Comparison of LFP change induced by sound between isoflurane and ketamine. Significant reductions of change were observed in broadband LFP (12 kHz 10%: *n* = 13, *N* = 3 in isoflurane, *n* = 9, *N* = 2 in ketamine; 12 kHz 50%: *n* = 18, *N* = 3 in isoflurane, *n* = 8, *N* = 2 in ketamine) (data expressed as mean ± SEM, statistics: Mann–Whitney test in (**c**, **g**), Multiple unpaired *t* test in (**d**, **h**). *P < 0.05, **P < 0.01, ***P < 0.001).

Under ketamine anesthesia, responses to ultrasound in the SC were entirely suppressed. This was evident in the absence of Ca^2+^ signal increases during 250 ms TBD and CW exposures (Fig. 5a, b). AUC quantification revealed significantly lower responses compared to those under isoflurane anesthesia (Fig. 5c) (Mann–Whitney test). The one-sample Wilcoxon test also showed that AUCs during ultrasound exposures were not significantly different from the theoretically expected “no change” response. To ensure that the signal noise in ketamine did not impact data interpretation, we performed an additional analysis by subtracting the AUC of the 20 s before exposure from that during ultrasound exposure. The results of this analysis were consistent with the absolute AUC analysis. Additionally, we investigated LFP changes under ketamine. In contrast to isoflurane, where 250 ms TBD ultrasound induced an overall increase in power, no discernible change was observed under ketamine (Supplementary Fig. 9) (one-way ANOVA and Dunnett’s multiple comparison test). Significant differences were identified in the delta, theta, and low-gamma frequency bands when comparing LFP changes in isoflurane and ketamine (Fig. 5d) (unpaired *t* test).

Next, we assessed the effect of ketamine on SC responses to sound. Ketamine completely blocked Ca^2+^ responses evoked by 10% duty cycle sound, though partial responses to 50% duty cycle sound remained (Fig. 5e–g). This was confirmed both in comparison to responses under isoflurane (Fig. 5g) and theoretical “no change” (*P* = 0.6257 for 10% and *P* < 0.05 for 50%, one-sample Wilcoxon test). Subtracting the baseline did not alter the results. However, no significant change in LFP was induced by sound under ketamine (Supplementary Fig. 10), which was different from the observations under isoflurane (Fig. 5h). A closer examination of the Ca^2+^ responses in isoflurane and ketamine revealed substantial differences in their dynamics (Supplementary Fig. 10f, e). Ca^2+^ responses in isoflurane exhibited an apparent peak at the onset of exposure and a rapid decay, while in ketamine, the peak response disappeared, and a sustained lower response persisted throughout the 20-s sound exposure. Statistical comparison of AUCs in isoflurane and ketamine also demonstrated a significant difference in the first second but not the subsequent 19 s (Supplementary Fig. 10f). Taken these data together, it is reasonable to conclude that ketamine altered the responses to sound in the SC.

### Ketamine blocks neuronal responses to ultrasound but not to sound in the auditory cortex

**Fig. 6:**
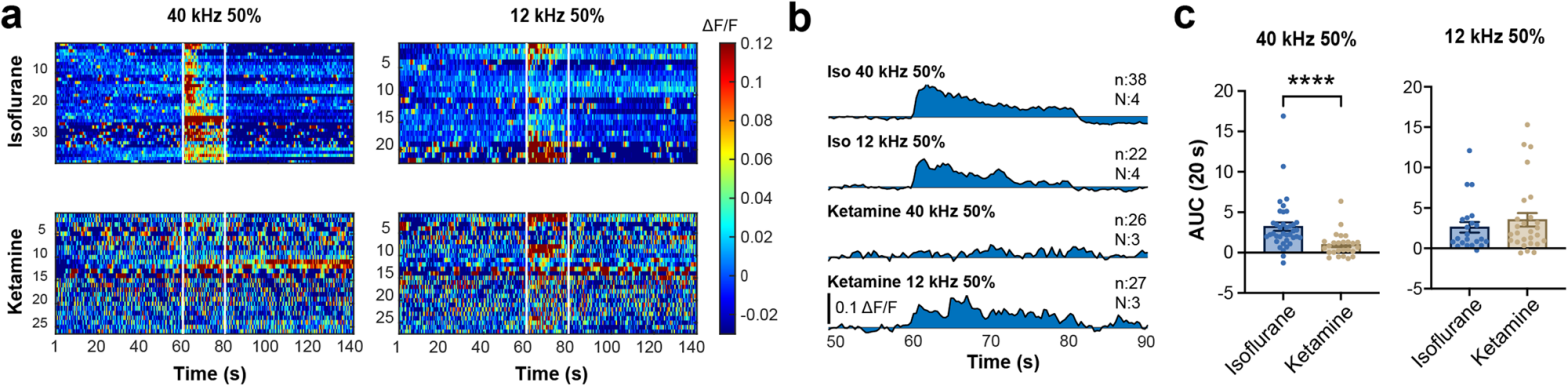
Ketamine blocked neuronal responses to ultrasound, not to sound in the auditory cortex. **a** Heatmaps showing Ca^2+^ responses to 50% duty cycle 40 kHz ultrasound and 12 kHz sound under isoflurane and ketamine anesthesia. **b** Average Ca^2+^ traces in response to 50% ultrasound and sound under isoflurane and ketamine. Note that no apparent increase in Ca^2+^ signals during ultrasound exposure under ketamine, while sound responses remained (*n* trial number, *N* animal number). **c** Comparison of AUC during 20-s ultrasound and sound exposures between isoflurane and ketamine. Significantly lower AUCs for ultrasound in ketamine were observed, whereas no significant difference in AUCs during sound exposure between isoflurane and ketamine (data expressed as mean ± SEM, Statistics: Mann–Whitney test in (**c**). ****P < 0.0001).

The inhibitory effect of ketamine observed in the SC prompted us to investigate its impact on the AC. Previous research has indicated that ketamine can alter specific features of auditory responses, although it may have no effect or enhance responses to pure tone sound stimulation^34–36^. If ultrasound and sound share the same pathway for inducing neuronal responses in the SC, one might expect that ketamine would have similar effects on responses to ultrasound and sound in the AC. However, our experiments uncovered that ketamine blocked Ca^2+^ responses to ultrasound in the AC but had no notable impact on responses to sound (Fig. 6a, b). This is evident from the significant reduction in the AUC during ultrasound exposure in ketamine, while there was no significant difference in AUCs during sound exposure between isoflurane and ketamine (Fig. 6c). These results suggest that although responses to ultrasound and sound in the SC and AC share similarities, they may involve different underlying mechanisms.

### Safety of transcranial application of 40 kHz ultrasound at 300 kPa

Low frequency ultrasound is known for its greater propensity to induce cavitation and sonoporation, resulting in increased cell membrane permeability^37^. While not serving as a comprehensive safety assessment, we performed astrocyte (GFAP), microglia (Iba-1) and neuronal (NeuN) staining on brain slices from animals exposed to ultrasound. Six months after a single 10% 500 µs exposure, we observed no evident glia reactions or neuronal damage compared to sham control (Supplementary Fig. 11). Moreover, during real-time Ca^2+^ imaging, we did not detect any immediate neuronal cell death or prolonged neuronal activation with any of the parameters used. However, it is essential to note that these findings cannot be extrapolated to ensure the safety of longer-term or higher-intensity applications. In addition, as we have previously reported, histology may not be the most sensitive method for detecting adverse event of ultrasound^38^.

## Discussion

Our study demonstrates that, similar to higher frequency ultrasound, 40 kHz ultrasound induces widespread neuronal activity in the cortical regions, including somatosensory and auditory cortices, through direct coupling. These responses depend on burst duration and resemble audible sound-induced responses. The in vitro brain slice experiments showed no neural responses to 300 kPa 40 kHz ultrasound, suggesting that a network presence is required for these responses. Ketamine, an NMDA receptor blocker, completely inhibits neural responses to ultrasound in both somatosensory and auditory cortices and partially affects sound responses in the somatosensory cortex but has no impact on auditory cortex audible sound responses. This implies that the cortical effects of low-frequency ultrasound heavily depend on NMDA receptors and may involve mechanisms beyond or different from indirect auditory cortex activation.

Ultrasound neuromodulation has been explored for treating neurological conditions, including chronic pain, epilepsy and psychological conditions^39–41^, and for enhancing cognitive and behavioral performance in healthy individuals^42^. Currently, the prevalent ultrasound frequencies for neuromodulation range from 200 kHz to 1 MHz, striking a balance between transcranial attenuation and focus size. Lower frequency ultrasound, despite advantages like reduced transcranial attenuation and better air transduction, has seen limited study in neuromodulation due to the challenges of concentrating energy into a small target and the higher risk of cavitation. However, the development of ultrasound multi-element arrays has made it possible to focus high-energy levels up to 160 dB within an area of about 1 cm^2^ (see ref. ^25^), making targeted transcranial applications feasible.

Several studies have suggested that low-frequency ultrasound could impact cognition and behavior^7,8^. In this investigation, we delved into the brain’s response to low-frequency ultrasound exposure in real-time and at both cellular and population level. Indeed, we observed both increased Ca^2+^ responses in individual excitatory neurons and elevation of broadband LFP, with a particularly pronounced increase in higher frequency oscillations, such as high-gamma band. Gamma oscillations are widely recognized for their critical roles in information processing and cortical arousal^43,44^. Alterations in gamma oscillations have been documented in multiple cognitive disorders^45^. The modulation of gamma oscillations by ultrasound suggests its potential as a therapeutic avenue for the treatment of such cognitive disorders.

Although mice may not be an optimal model for 40 kHz ultrasound, given that it falls within their audible range, and our study did not block the hearing pathway, we found that air-transduced ultrasound elicits minimal activity in the auditory cortex compared to direct coupling and 12 kHz sound. Furthermore, ketamine specifically blocked the ultrasound response in the auditory cortex, leaving the audible sound response unaffected. These findings imply that the observed ultrasound effects in our study do not follow the conventional pathway of air-transduced sound detection.

However, previous research has demonstrated ultrasound-induced widespread neuronal activity in the cortex. Removing cochlear fluid or chemically inducing deafness in the inner ear can eliminate these responses^18,19^, suggesting that the widespread excitation likely results from the propagation of activations from the auditory cortex through cortico-cortical interactions. It is unclear whether 40 kHz ultrasound shares the same activation mechanism as higher-frequency ultrasounds tested in those prior studies. If it does, the divergence between ultrasound and sound response pathways likely occurs downstream of the inner ear, since it is known that NMDA receptors play little role in the inner hair cell and spiral ganglion synapses^46^. The precise pathway warrants further investigation in future studies.

In addition to the activation of auditory pathway, studies on cell culture and brain slices have shown direct activation of neurons^16,33^. Also, it was suggested that astrocyte may have a lower activation threshold, which release glutamate and excite nearby neurons through NMDA receptors^17^. However, our in vitro study did not show responses to 40 kHz ultrasound, while robust responses were observed with 500 kHz ultrasound. Therefore, the astrocyte pathway cannot explain our observations with ketamine. One possible explanation for the ineffectiveness of the 40 kHz ultrasound could be the difference in waveforms produced by the transducers. The 40 kHz transducer generates a planar wave, whereas the focused 500 kHz transducer produces a focused ultrasound. The acoustic-brain interface experiences increased shear forces with the 500 kHz frequency. The minimal shear forces generated by the planar wave of the 40 kHz transducer could contribute to its lower effectiveness compared to the 500 kHz ultrasound. Notably, the presence of standing wave may substantially reduce or eliminate the pressure on the slice, which has been reported in other in vitro studies. The current set up has ~1 mm ultrasound gel, ~3 mm ACSF solution and 0.14 mm cover glass between the transducer cone and brain slice, and the wavelength of 40 kHz is estimated to be 37.5 mm in gel/water, 141 mm in glass and 38.5 mm in brain tissue, which is up to two orders longer than the medium thickness. For a standing wave to form effectively, a medium’s thickness often needs to be comparable to a half-wavelength or a multiple of half-wavelengths of the sound wave. Given the mismatch in the dimensions and wavelengths in our setup, standing waves are less likely to be sustained. On the other hand, the wavelength for 500 kHz is around 3 mm in gel/water, 11.28 mm in glass and 3.08 mm in brain, which is more likely to form standing wave. However, given the result that 500 kHz induced stronger neural activity in our in vitro study, we expect the standing wave for 500 kHz did not remarkably reduce the pressure in the tissue. Overall, regardless of the underlying reasons for its ineffectiveness in inducing neuronal activity in vitro, this suggests that our observed cortical effects result from a network effect.

The involvement of neural networks in low-frequency induced neuronal activity can also provide a plausible explanation for the observed disparity between Ca^2+^ signal and LFP analyses. In our Ca^2+^ analysis, CW exposures induced the highest magnitude of responses in individual excitatory neurons, whereas 250 ms pulse produced the most prominent response in LFP, with less effect from CW. Unlike Ca^2+^ imaging, which reveals activity at the level of individual neurons, LFP reflects ensemble activities of all types of neurons. Several preliminary investigations have demonstrated diverse responses of various cell types to different ultrasound parameters. For example, Manuel et al. reported that CW was less effective in inducing Ca^2+^ responses in brain slices, without considering cell type^28^. Yu et al. and Murphy et al. showed different types of neurons exhibited varying responses to different ultrasound parameter sets^29,30^. Furthermore, correlation patterns between single unit activity and LFP oscillations vary among cell types and cortical status^31,43^. These findings underscore the complexity of neuronal responses to ultrasound stimulation and highlight the importance of considering the diversity of neural populations and their interactions within neural networks when interpreting experimental results. This complexity warrants further investigation into the mechanisms underlying the neural effect of low-frequency ultrasound.

The absence of neuronal response in brain slice preparation may raise concerns about the confounding effect of sensory perception, such as mechanical vibration or heating. However, our observation that ketamine effectively blocked the ultrasound-induced response suggests that the involvement of peripheral sensation response is likely limited. This interpretation is supported by prior studies demonstrating that ketamine does not diminish sensory-evoked cortical responses^47,48^. We also believe the involvement of nociceptive pathway is unlikely in our observation. Our intracranial temperature measurement revealed less than 1 °C temperature rise. And the temperature measurement against the skull wall demonstrated a maximum 1.15 °C increase. These temperature changes were well below the threshold for inducing thermal pain sensation^49^.

Low-frequency ultrasound is commonly employed for device cleaning due to its ability to generate cavitation^50^. Given that we implanted an electrode into the somatosensory cortex for electrophysiological recording, it is important to consider whether the electrode’s vibration might impact the interpretation of the observed neural effects. However, considering the striking similarity of ultrasound-induced neuronal effects to those elicited by sound in both the auditory and somatosensory cortices, as well as the complete abolition of ultrasound effects by ketamine, we believe that any potential confounding effect of electrode vibration on our observations is minimal, if not non-existent.

Another limitation of this study is the presence of an unblocked auditory pathway. Given that our research involved major survival surgery and repeated experiments in live animals, interventions such as chemical or surgical procedures to disrupt the auditory system would have negatively impacted the animals’ recovery. The results of this study could be validated in the future using alternative animal models or genetically deaf mouse strains.

## Methods

### Experimental model and study participant details

All animal use procedures in this study were approved by the Food and Drug Administration (FDA) White Oak Institutional Animal Care and Use Committee (WO IACUC) and Animal Care and Use Review Office (ACURO) of the Department of Defense. All procedures complied with the National Institutes of Health Guide for the Care and Use of Laboratory Animals. Adult male Thy1-GCaMP6s transgenic mice (C57BL/6J-Tg (Thy1-GCaMP6s)GP4.3Dkim/J, Jackson Laboratory, Bar Harbor, Maine, Stock No: 024275) aged 2–6 months were used for calcium imaging.

### Surgery

During surgery, mice were anesthetized with a 4% induction dose of isoflurane (Henry Schein, Melville, New York), then positioned in a stereotaxic apparatus (David Kopf Instruments, Tujunga, California). Mice were maintained under anesthesia with ~1.5% isoflurane (0.8 L ∕min O_2_), body temperature was maintained at ∼37 °C with a thermostat-controlled heating plate (Model TC-1000, CWE Inc., Ardmore, Pennsylvania), and respiration rate was monitored and maintained at ∼100 breaths/min during the procedure.

To evaluate the ultrasound effect on the somatosensory cortex, a craniotomy (∼2 × 3 mm) was created over the left somatosensory cortex (coordinates relative to Bregma in mm: AP −0.5 to −3.5, L 0.5 to 2.5) using a high-speed dental drill (Osada, 0.25-mm drill bit, Osada, Inc Los Angeles, California) as previously reported^51^. A custom-cut 2 × 2 mm glass coverslip (sterilized #0) was placed on the surface of the cortex above the dura and attached to the skull with Kwik-Sil (WPI, Sarasota, Florida) and dental cement (Parkell C&B Metabond, Edgewood, New York). A metal bar with a screw notch was attached over the skull on the right hemisphere to stabilize the head during in vivo imaging. This preparation left an unobscured imaging area of ∼1.5 × 1.5 mm. For simultaneous electrophysiological recording, a single-shank, 16-channel microelectrode array (A1×16-3mm-100-703-CM16LP, NeuroNexus, Ann Arbor, Michigan) was inserted into the cortex under the glass window with a 10–20° angle relative to the brain surface through the posterior edge of the craniotomy. The electrode has 16 iridium recording sites sized 703 μm^2^ that were linearly distributed along the shank (15 μm thick and 123 μm maximum width) with 100 μm spacing between sites. The tip of electrode array ended at about 200–300 µm deep.

For auditory effect recording, the similar window implantation approach was used. However, the craniotomy was a 3 mm diameter round shape with the center at the approximate coordinates of AP −3 mm and ML 4 mm at either the left or right hemisphere. A 3 mm diameter #0 thickness cover glass (CS-3R-0, D263 Coverslip/Coverglass, Warner Instrument) was placed on the surface of the cortex. No electrode was implanted in the auditory cortex. A metal bar was attached on the skull on the other hemisphere with a bended angle at about 45°.

Two photon microscopy (TPM) imaging and electrophysiological recordings were performed no earlier than 1 day after surgery. When comparing the effects of ketamine and isoflurane, animals were imaged and recorded on separate days, with a minimum 24-h recovery period between experiments.

### Ultrasound exposures

The ultrasound system was composed of a power amplifier (150A100B, Amplifier Research, USA), a function generator (FG 3102 C, Tektronix), and a bolt clamped Langevin 40 kHz transducer (SMBLTD45F40H, Steiner & Martins, Inc., USA) with a spatial fitting cone composed of nylon material acoustic matching layer (Fig. 1a). The matching cone has a height of 9 cm and a tip diameter of 1 cm in order to spatially fit the in vivo imaging setup. For in vivo experiment, the transducer was placed against the temporal side of the unshaved mouse head with a thin layer of ultrasound gel for coupling (Fig. 2a). For in vitro brain slice experiment, the transducer was placed under the slice chamber. The bottom of the slice chamber was a #1 glass cover slip. A thin layer of ultrasound gel was applied between the transducer tip and cover slip. In the in vitro brain slice calcium imaging experiment, a 500 kHz ring-geometry transducer (H107, Sonic Concept) with a home-built 2% Agar-based coupling cone was also used to compare results with the 40 kHz transducer. The output of the 500 kHz ultrasound was calibrated in a degassed water tank with the presence of cone, using the needle hydrophone (NH1000, Precision Acoustics, UK).

Ultrasounds were applied as pulses or continuous waves. Altogether, seven paradigms of 40 kHz ultrasound were tested as listed in Table 1. The spatial and temporal profile of the pressure was characterized in a degassed water tank with a needle hydrophone (NH1000, Precision Acoustics, UK).

### In vivo two-photon microscopy (TPM) imaging

Animals were imaged on a Movable Objective TPM Microscope (MoM, Sutter Instrument) with MScan 2.0 software. Mice were anesthetized with a 4% induction dose of isoflurane and then maintained with 1% to 2% of isoflurane throughout imaging. In a subset of animals, ketamine/xylazine cocktail (1.5 mg/20 g Ketamine and 0.3 mg/20 g xylazine) was administrated every 30–60 min according to respiration rate and/or electrophysiological signals. Body temperature was maintained at 37 °C on a heating pad with a closed-loop thermostat-controlled heating plate (Model TC-1000, CWE Inc., Ardmore, Pennsylvania), and respiration rate was monitored and maintained at ∼100 breaths/min during imaging. The animal was positioned for TPM imaging using a three-axis motorized animal stage (AS) (Thorlabs Inc., Newton, New Jersey). For each ultrasound exposure trial, a 60 s baseline, 20 s ultrasound exposure and 60 s of post-exposure calcium signals were acquired at a frame rate of 3. Multiple regions of interest (ROIs) were located for imaging in each animal. Multiple ultrasound dosages and sham control were delivered in a random order to each animal in each ROI to avoid region, anesthesia duration, and exposure sequency bias. Between each exposure, 5 mins of interval was applied. The dosage delivery order was also randomized between animals. Each imaging experiment lasted from 1.5 to 3 h.

### Brain slice preparation and in vitro imaging

Acute brain slices were prepared from 2 to 6-month-old Thy1-GCaMP6s mice as described in ref. ^52^. After anesthetization with 200 mg/kg sodium pentobarbital, confirmed with no toe pinch reflex, the animal was decapitated. After dissection, the brain was transferred and immersed in ~4 °C oxygenated (95% O_2_/5% CO_2_ mix) sucrose-substituted artificial cerebrospinal fluid (aCSF) for slice preparation. The sucrose aCSF was composed of 210 mM Sucrose, 26 mM NaHCO_3_,8 mM MgCl_2_, 0.5 mM CaCl_2_, 10 mM HEPES, 20 mM glucose, 0.4 mM ascorbic acid. Coronal slices were cut to a thickness of 300 µm (7000 smz vibrotome, Campden Instruments). After the cutting procedure, slices were incubated in oxygenated regular aCSF at room temperature for at least 30 min before imaging. The regular aCSF consisted of 117 mM NaCl, 4.7 mM KCl, 1.2 mM MgCl_2_, 2.5 mM CaCl_2_, 1 mM NaH_2_PO_4_·H_2_O, 24 mM NaHCO_3_, 10 mM glucose, 5 mM HEPES. After incubation, slices were moved to the chamber maintained between 33 °C and 36 °C with consistent perfusion of oxygenated aCSF at 3–5 ml/min rate. Calcium imaging was performed using an upright Zeiss AxioExaminer D1 microscope (Carl Zeiss Microscopy GmbH) at about 6 frames/s with Zen 3.4 Pro software (Carl Zeiss Microscopy GmbH).

### In vivo electrophysiology data acquisition

Electrophysiological signals were acquired simultaneously with TPM calcium imaging via a Scout processor, Nano^2+^Stim front end and Trellis software (Ripple Neuro, Salt Lake City, UT) at a sampling frequency of 30 kHz from anesthetized mice. Raw and band-pass (0.5–300 Hz) filtered signals were saved after notch-filtering at 60, 120 and 180 Hz.

### Acoustic stimulation

The acoustic stimulation was performed through an acoustic speaker with a power amplifier (SERVO 120a, SAMSON, USA). Pure tones (12 kHz at 75 dB, 10% and 50% duty cycles were delivered through the free-field speaker coupled with a paper cone targeting the mouse ear at a distance around 1.5 cm. Due to the limitation of burst stability and maximum duty cycle, the actual output tone burst duration was 50–100 ms, with duty cycle of 4.3% and 33.3%.

### Calcium imaging analysis

After imaging acquisition, full field or individual neuronal activity was quantified by fluorescent calcium signals. calcium transients (ΔF/F) were quantified by the change in fluorescent intensity calculated as (F_t_-F_0_)/F_0_, where F_0_ is the 60 s baseline fluorescent intensity before ultrasound exposures. ImageJ was utilized for individual cell selection and integral fluorescent intensity determination. The analyzers were blinded to the dosage of the exposure while selecting individual cells with attempt to include all cells in the imaging field. MATLAB was used for further processing of calcium data, including event detection and area under the curve calculation. For event detection, a threshold of 1.5 × STD of the mean ΔF/F was used. Calcium signals of the top ten responding cells in each trial were auto-selected based on the calcium event number change. Group or dosage information associated with each calcium time series were blinded for calcium imaging analysis until statistical analysis stage.

### Electrophysiology data analysis

Band-pass (0.5–300 Hz) filtered local field potential (LFP) data from 16 channels were analyzed with MATLAB function *pspectrum* to calculate the power spectral density (PSD) of 20 s signals before, during and after ultrasound exposures. PSD change ratio was determined via dividing PSD during or after exposures by that of before exposure. Only data obtained from the deepest two channels from each array were included in the further statistical analysis. The power bands are defined as δ: 1–4 Hz, θ: 4–8 Hz, α: 8–13 Hz, β: 13–20 Hz, low-γ: 20–56 Hz, high-γ: 64–100 Hz.

### Quantification and statistical analysis

To compare calcium signals between multiple groups, nonparametric version of one-way ANOVA, Kruskal–Wallis test, was used. For multiple comparison correction, Dunn’s test was used to compare treated groups with the control group. To compare between two groups, Mann–Whitney test was used. For electrophysiological data, one-way ANOVA test and post-hoc Dunnett’s or Tukey test was used to determine differences between multiple groups, and for two group comparison, Student t test was used. Because LFP from normal subjects was better known, it is more reasonable to assume they are normal distributed. All statistics were conducted with GraphPad Prism 9.0 or 10.0. When *P* value is lower than 0.05, stars are used to indicate different levels of significance. When p value is between 0.05 and 0.1, the *P* value is provided in figures. When *P* value is larger than 0.1, no marks are labeled in figures.

### Reporting summary

Further information on research design is available in the Nature Portfolio Reporting Summary linked to this article.

### Supplementary information

Supplementary Information

Reporting Summary

## Data Availability

The original data reported in this paper will be shared by the corresponding author (Meijun.ye@fda.hhs.gov) upon request. This paper does not report original code. Any additional information required to reanalyze the data reported in this paper is available from the corresponding author upon request.
