# Supplementary material for: Low frequency ultrasound elicits broad cortical responses inhibited by ketamine in mice
Source: Commun Eng. 2024 Aug 27;3:120. doi: 10.1038/s44172-024-00269-2 (PMC11349898; doi:10.1038/s44172-024-00269-2)
Supplement: Supplementary file 1 — Supplementary Information [file 44172_2024_269_MOESM1_ESM.pdf]

Supplemental Information

Low frequency ultrasound elicits broad cortical responses inhibited by ketamine in mice

Authors: Linli Shi[1], Christina Mastracchio[1], Ilyas Saytashev [1], Meijun Ye[1,*]

Affiliations: 1. Division of Biomedical Physics, Office of Science and Engineering Laboratories, Center for Devices and Radiological Health, Food and Drug Administration

*. Corresponding author: meijun.ye@fda.hhs.gov

SUPPLEMENTARY METHODS

Intracranial ultrasound pressure measurement

For the intracranial pressure measurement, after euthanasia with overdose of sodium pentobarbital (390 mg/ml), the mouse was decapitated following hair removal with Nair. Mouse brain was removed from foramen magnum. Teledyne miniature hydrophone (RESON TC 4047, Teledyne Marine, USA) was placed within mouse cadaver heads in degassed water. Large tank and short burst (10 cycles) were used to avoid wall and surface reflections. Mouse head position was adjusted to find maximum signal while transducer and hydrophone were stationary. Signals were evaluated at the time of entry and after multiple reflections.

Intracranial ultrasound simulation

The 3D simulation of intracranial ultrasound pressure utilized the K-wave Toolbox version 1.3. MicroCT scans of the mouse head were obtained from the Open Science Framework (OSF) as open-source data. The skull was isolated through thresholding techniques. The ultrasound source was configured with a diameter of 10 mm. Based on the experimental data depicted in Fig 1, the pressure decays to 1/e at a distance of 8.4 mm. Consequently, a scattered ultrasound source was defined by designing a curved surface with a focal length of 13 mm to match the decay curve. The medium was characterized by an alpha power (y) of 1.5. For bone, the alpha coefficient was 8.83 dB/(MHz y cm), the sound speed was 2850 m/s, and the bulk density was 1732 kg/m^3. The background medium was specified as water with an alpha coefficient of 0.05 dB/(MHz y cm), a sound speed of 1482 m/s, and a density of 1000 kg/m^3. These settings were according to Fomenko, Chen, et al. (*1*).

Histology

Mice were deeply anesthetized with sodium pentobarbital (100 mg/kg, i.p.) and perfused transcardially with phosphate buffered saline (PBS) and then 10% formalin (Fisher Scientific, Hanover Park, Illinois). Brains were extracted and cut to 50 µm thick coronal slices using a vibrating microtome (Campden Instruments, Lafayette, Indiana). Coronal sections located at 0, -1, and -2 mm to the Bregma were immunostained. Free-floating sections were washed in 1× PBS and incubated in a 0.3% Triton X-100 and 4% normal goat serum (NGS, Millipore Sigma, St. Louis, Missouri) solution for 1 h at room temperature to permeabilize the membrane and block nonspecific binding. Slices were then incubated overnight on a shaker at room temperature with primary antibodies against NeuN (pChicken; Millipore Sigma) to stain neuronal cytoplasms, GFAP (mRat; ThermoFisher, Waltham, Massachusetts) for astrocytes, and Iba1 (pRabbit; Wako, Osaka, Japan) for microglia, each at 1:500 dilutions in 0.3% Triton X-100/ 4% NGS in 1× PBS solution. The following day, slices were washed in PBS, then incubated for 1 h at room temperature with corresponding secondary antibodies raised in goat: Alexa Fluor 405 (anti-chicken; Abcam, Cambridge, Massachusetts), Alexa Fluor 488 (anti-rat, ThermoFisher), and Alexa Fluor 647 (anti-rabbit, ThermoFisher), each at 1:1000 dilutions in PBS. Stained slices were mounted on gelatin subbed microscope slides and cover-slipped with Fluoromount (Southern Biotech, Birmingham, Alabama). Fluorescent images were obtained with an Olympus (Center Valley, Pennsylvania) FV3000 confocal microscope.

Supplementary Figure 1

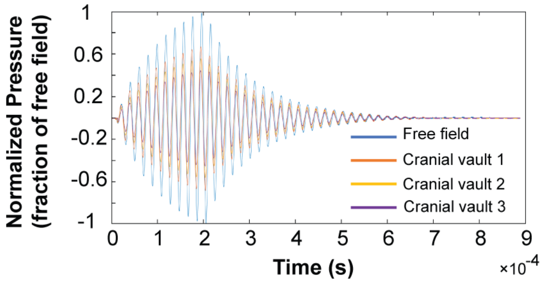

Supplementary Figure 1. Normalized intracranial pressure compared to the free field. 60-90% transmission rate through the skull at the unreflected entry pulse was measured, while the peak reflected pressure was 40-70% of the free field.

Supplementary Figure 2

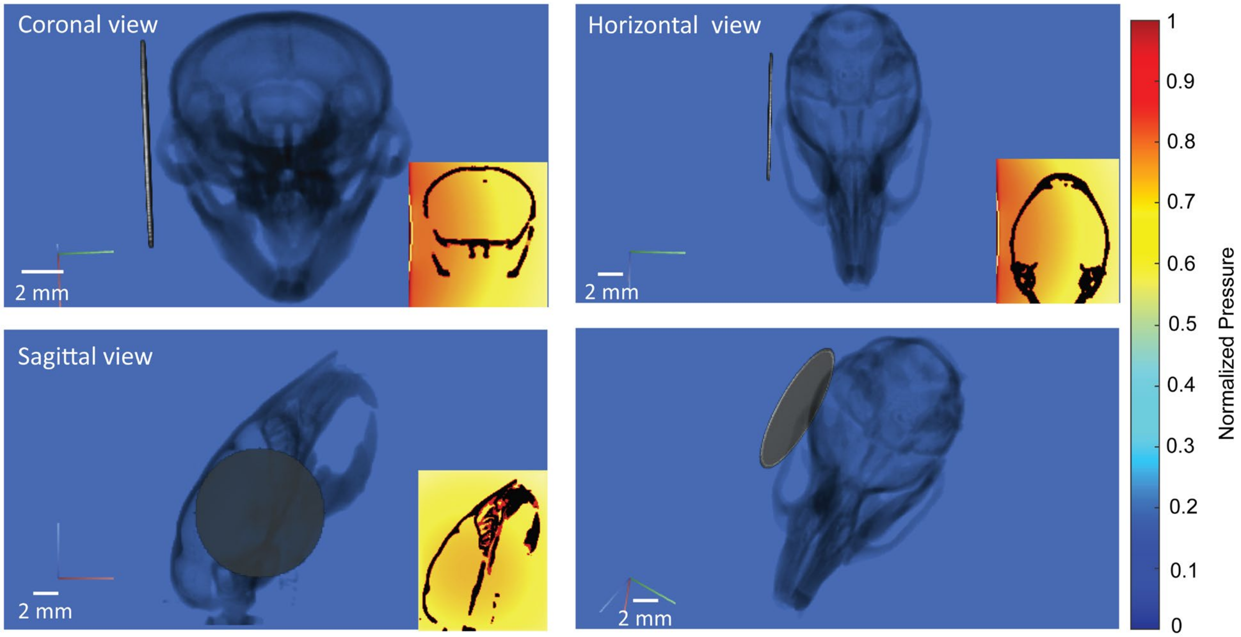

Supplementary Figure 2. 3D simulation of the acoustic propagation through the mouse skull. A 60-80% transmission rate was revealed.

Supplementary Figure 3

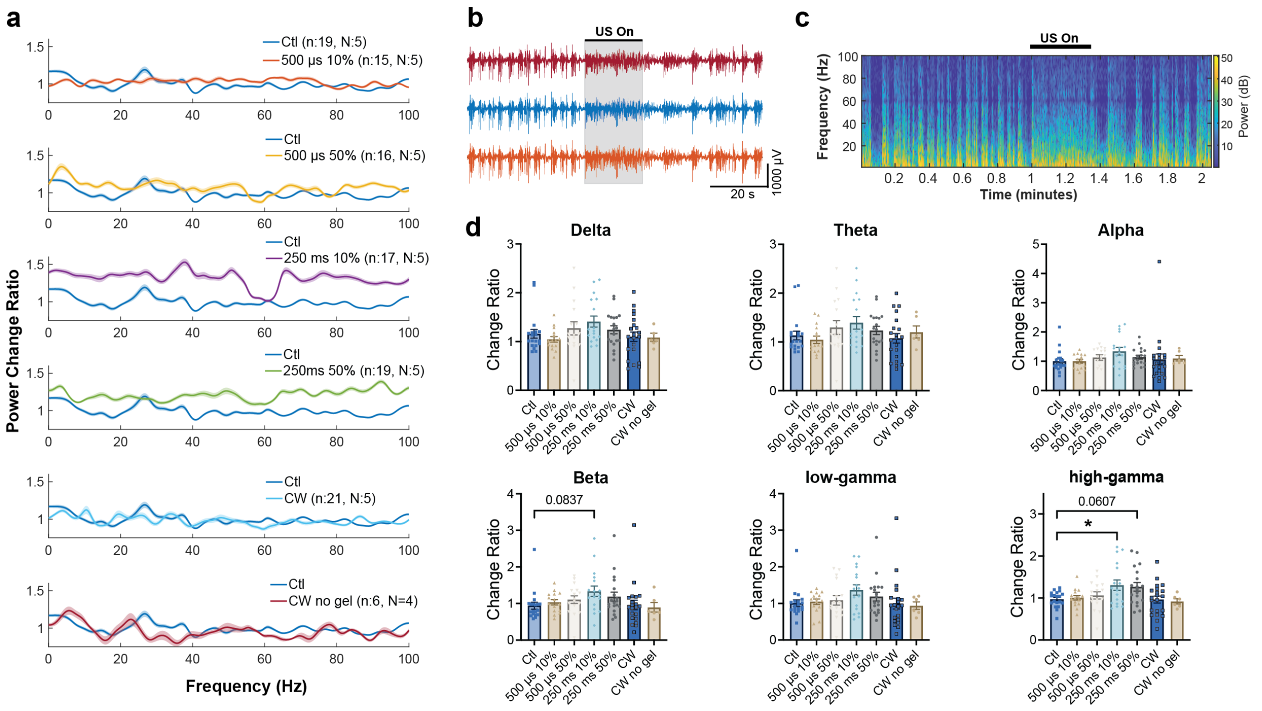

Supplementary Figure 3. Ultrasound exposure altered local field potential (LFP) of the somatosensory cortex. (a) Power spectral density (PSD) change ratio of the LFP during ultrasound exposure. Note the increase in the overall power during 250 ms pulse exposures. Data expressed as Mean ± SEM (n: trial number, N: animal number). **(b)** Example LFP traces from three channels. Note the overall oscillation change during a 250 ms 50% duty cycle ultrasound exposure indicated with gray bar. **(c)** The time-frequency spectrogram of the bottom LFP signals in (b). Note the increase in the power of overall frequency band during ultrasound exposure indicated by black bar on the top. **(d)** Summary of PSD change in each frequency band. Each dot represents each trial. Data expressed as Mean ± SEM. Statistics: One-way ANOVA with Dunnett's multiple comparison test. * $p < 0.05$.

Supplementary Figure 4.

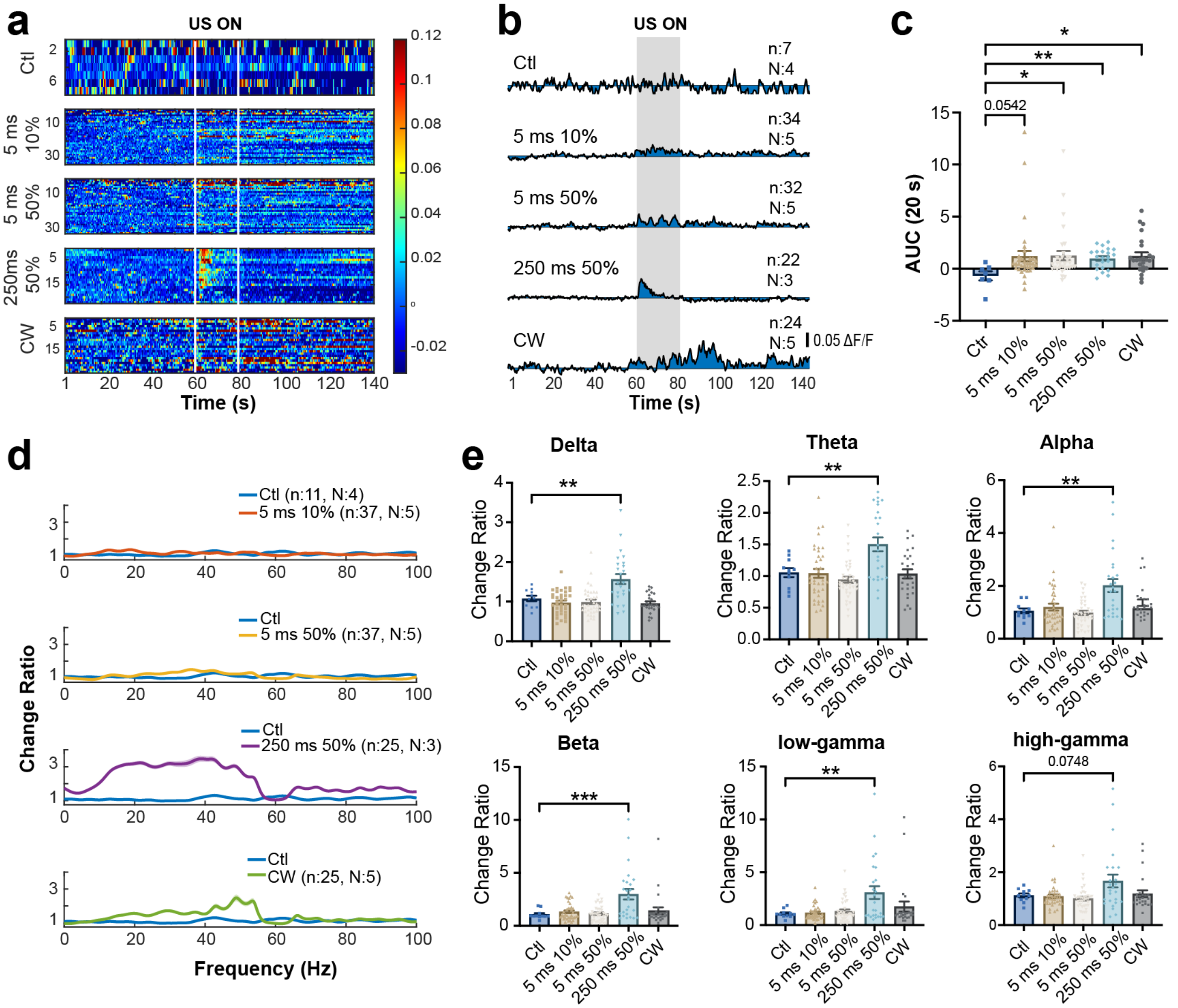

Supplementary Figure 4. Ultrasound exposure increased neuronal activity at the somatosensory (SC) cortex in vivo. (a) Heatmaps of Ca^{2+} signals of the average of top ten responding cells in each trial for each parameter set in ΔF/F. **(b)** Traces of the mean Ca^{2+} signal of all trials for each parameter set in the heatmap, which demonstrates a clear parameter and response relationship (n: trial number; N: animal number). **(c)** Statistical summary of area under the curve (AUC) of Ca^{2+} signals during ultrasound exposure for each parameter set. Data expressed as Mean ± SEM, and each dot represents each trial. (Kruskal-Wallis and Dunn's multiple comparison test. * $p < 0.05$, ** $p < 0.01$). **(d)** Power spectral density (PSD) change ratio of the local field potential (LFP) during ultrasound exposure. Note the increase in the overall power during 250 ms pulse exposures. Data expressed as Mean ± SEM (n: trial number, N: animal number). **(e)** Summary of PSD change in each frequency band. Each dot represents each trial. Data expressed as Mean ± SEM. Statistics: One-way ANOVA with Dunnett's multiple comparison test. * $p<0.05$, ** $p < 0.01$, *** $p < 0.001$.

Supplementary Figure 5

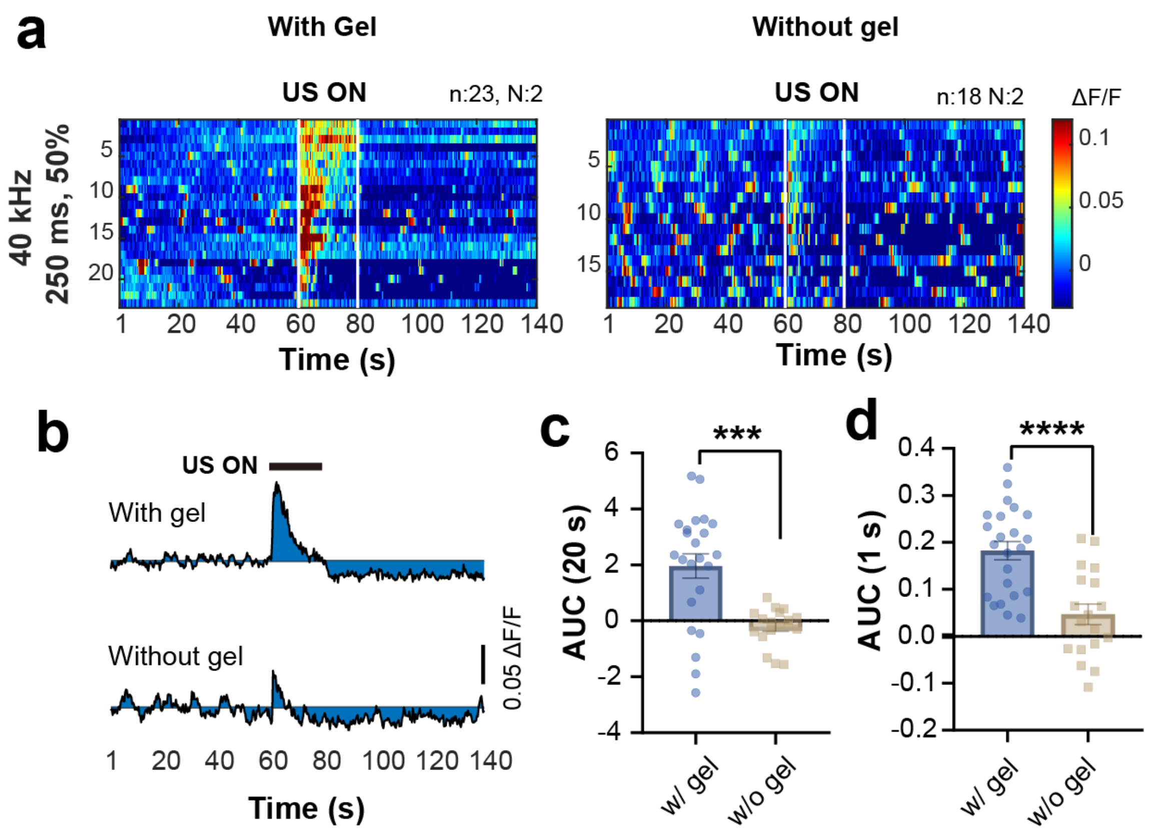

Supplementary Figure 5. Removing the direct coupling between transducer and head significantly reduced responses to 40 kHz ultrasound in the auditory cortex. (a) Heatmaps of Ca^{2+} signals of the average of top ten responding cells in each trial for conditions with and without ultrasound gel (n: trial number; N: animal number). **(b)** Traces of the mean Ca^{2+} signal of all trials for each condition, which demonstrates a clear reduction in response after removing the gel. **(c)** Statistical summary of area under the curve (AUC) of Ca^{2+} signals during 20 second ultrasound exposure for each condition. **(d)** AUC of the 1st second Ca^{2+} responses. Data expressed as Mean ± SEM in (c) and (d), and each dot represents each trial. (Statistics: Mann-Whitney test. *** $p < 0.001$, **** $p < 0.0001$)

Supplementary Figure 6

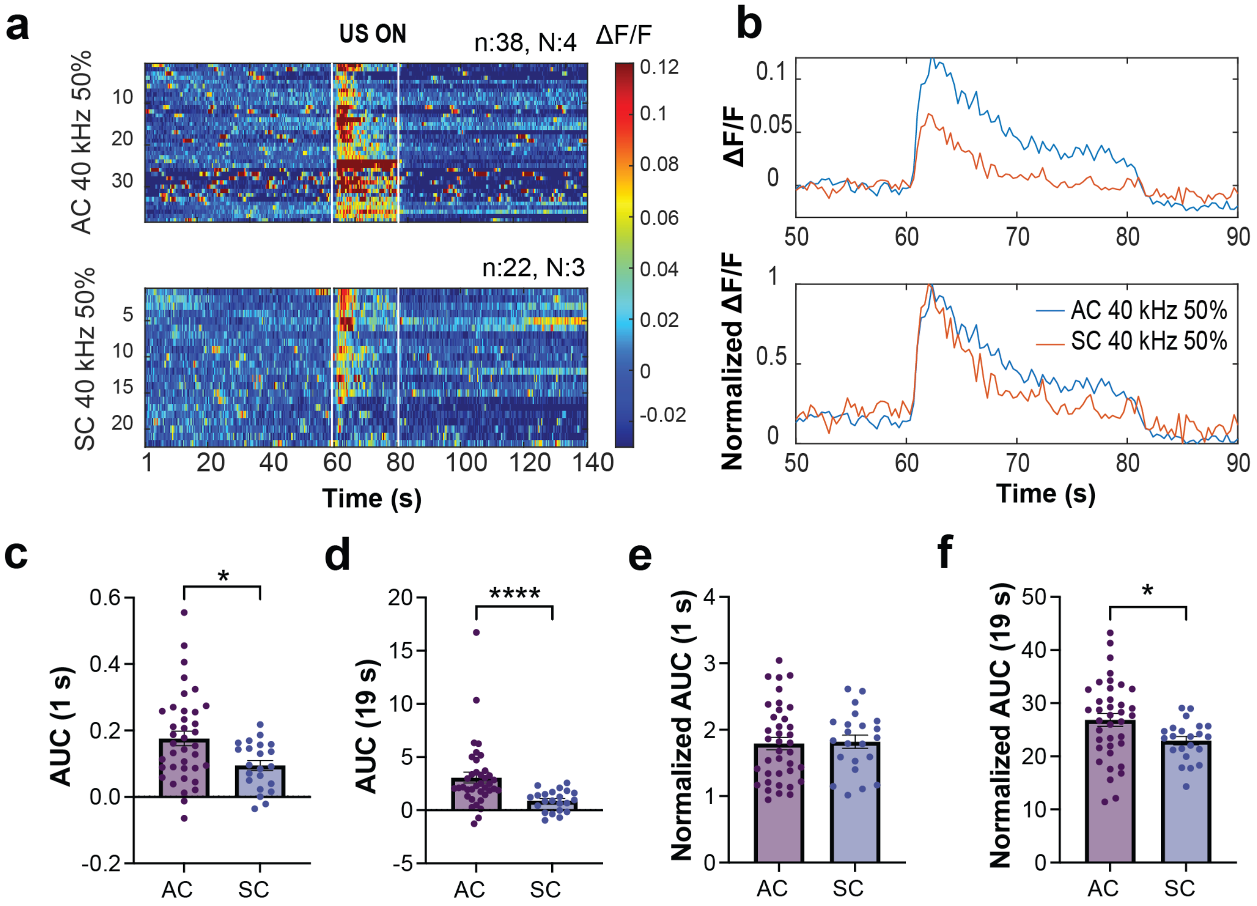

Supplementary Figure 6. Lower magnitude and faster decay of Ca^{2+} responses to 40 kHz ultrasound in the somatosensory cortex compared to those in the auditory cortex. (a) Heatmaps of Ca^{2+} signals in response to 50% 250 ms TBD ultrasound in the auditory cortex (AC) and somatosensory cortex (SC) respectively. **(b)** Comparison of the dynamics of Ca^{2+} responses to 50% 250 ms TBD ultrasound in the AC and SC. The top panel shows raw ΔF/F, suggesting a higher response in the AC compared to the SC. The bottom panel shows the trace normalized to the peak ΔF/F, indicating that both AC and SC exhibit a similar onset response, but the response in the AC decays more slowly. **(c)** Summary of the area under the curve (AUC) for the first second of response. AUC values in the SC were significantly smaller than in the AC. **(d)** AUCs for the 2 to 20-second of the Ca^{2+} signal. AUCs in the SC were significantly smaller compared to the AC. **(e)** No significant difference in the normalized 1st-second AUC between the AC and SC, suggesting a similar response speed. **(f)** Significantly smaller normalized AUC of 2 to 20-second in the SC compared to the AC, indicative of a faster decay. (Statistics: Mann-Whitney test in (c) to (f). * $p < 0.05$, **** $p < 0.0001$. Data are expressed as Mean ± SEM, and each dot represents one trial. n: trial number; N: animal number.)

Supplementary Figure 7

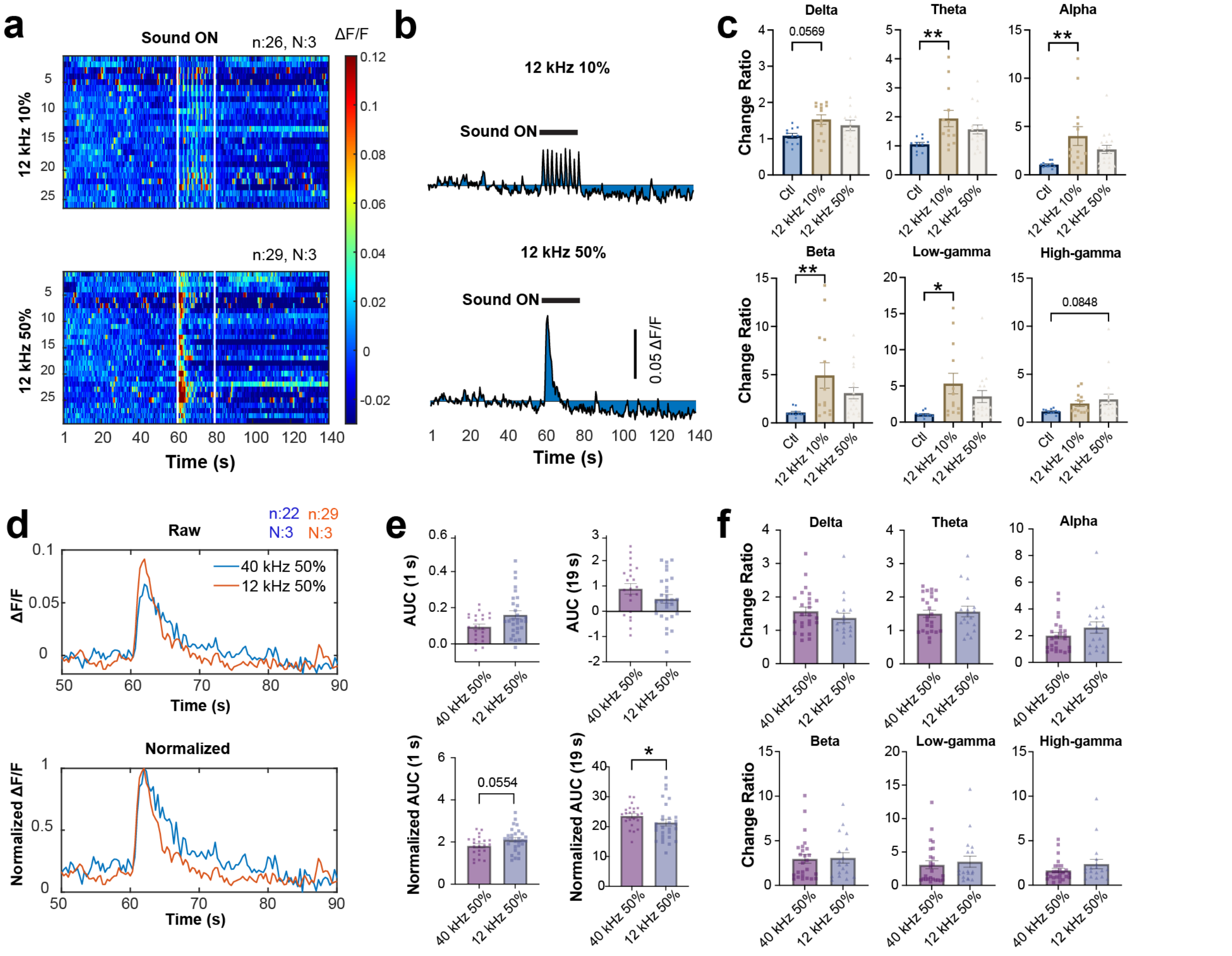

Supplementary Figure 7. Comparable magnitude but slower decay of Ca^{2+} responses to ultrasound compared to 12 kHz sound in the somatosensory cortex. (a) Heatmaps showing Ca^{2+} responses to 10% and 50% duty cycle 12 kHz sound at 75 dB. **(b)** Average Ca^{2+} traces in response to 10% and 50% 12 kHz sound, respectively. **(c)** Local field potential (LFP) changes during sound exposures. Increased overall power was detected for both 10% and 50% sound exposures. Quantification suggests significant increase in theta, alpha, beta, and low-gamma bands for 10% duty cycle (Ctl: n = 11, N = 4; 12 kHz 10%: n = 13, N = 3; 12 kHz 50%: n = 18, N = 3). **(d)** Comparison of Ca^{2+} traces in response to 50% of 40 kHz ultrasound and 12 kHz sound. Top panel shows the raw traces, and the bottom panel presents the normalized traces. **(e)** Amplitude and dynamic comparison of Ca^{2+} responses to 50% 40 kHz ultrasound and 12 kHz sound. Area under the curve (AUC) of the 1st and 19 second (2-20 second after the onset of exposure) demonstrated no significant difference between the two groups, suggesting comparable magnitude of responses. However, nonsignificant higher 1st-second normalized AUC and significant lower later 19-second normalized AUC for the 12 kHz group indicate that response to sound has a faster decay compared to ultrasound. **(f)** No significant difference in the LFP changes between ultrasound and sound (40 kHz 50%: n = 25, N = 3; 12 kHz 50%: n = 18, N = 3). (Data expressed as Mean ± SEM, Statistics: One-Way ANOVA and Dunnett's multiple comparison tests in (c), Mann-Whitney test in E, and unpaired t-test in F. * $p < 0.05$, ** $p < 0.01$)

Supplementary Figure 8

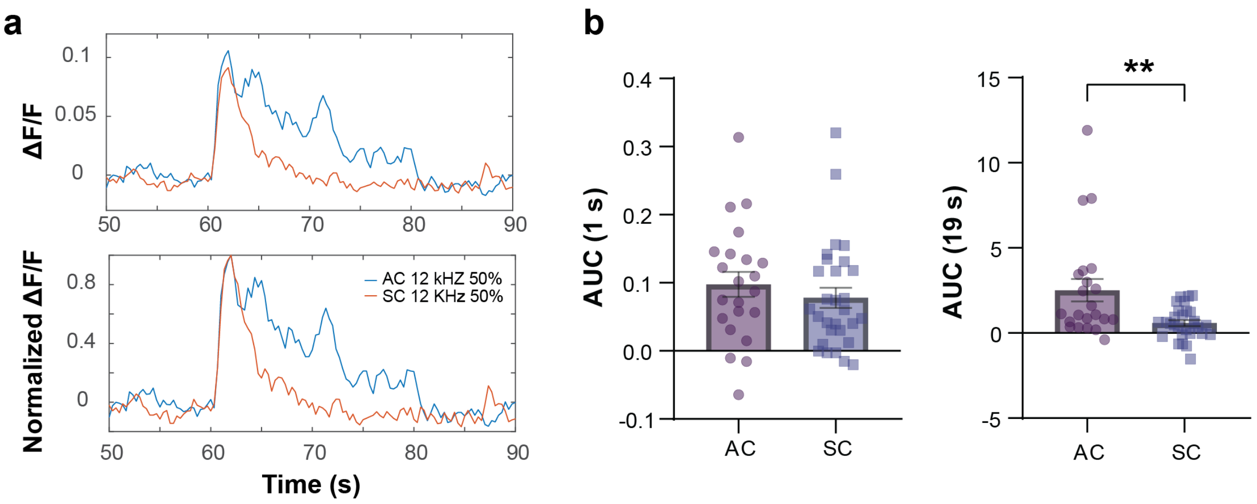

Supplementary Figure 8. Same onset but faster decay of Ca^{2+} responses to 12 kHz sound in the somatosensory cortex compared to those in the auditory cortex. (a) Comparison of Ca^{2+} responses to 50% sound in the auditory cortex (AC) and somatosensory cortex (SC). Top panel shows the raw ΔF/F. Bottom panel shows the trace normalized to the peak ΔF/F, indicating AC and SC have the same onset response, while response in AC has a slower decay. **(b)** Summary of the area under the curve (AUC) of the first second and during 2 to 20 second of the Ca^{2+} signal. AUCs of the first second in SC were not significantly different from in the AC. However, AUCs of the later 19 second in the SC were significantly smaller than in the AC, indicative of faster decay. (Statistics: Mann-Whitney test in (b). ** $p < 0.01$). Data are expressed as Mean ± SEM, and each dot represents one trial.

Supplementary Figure 9

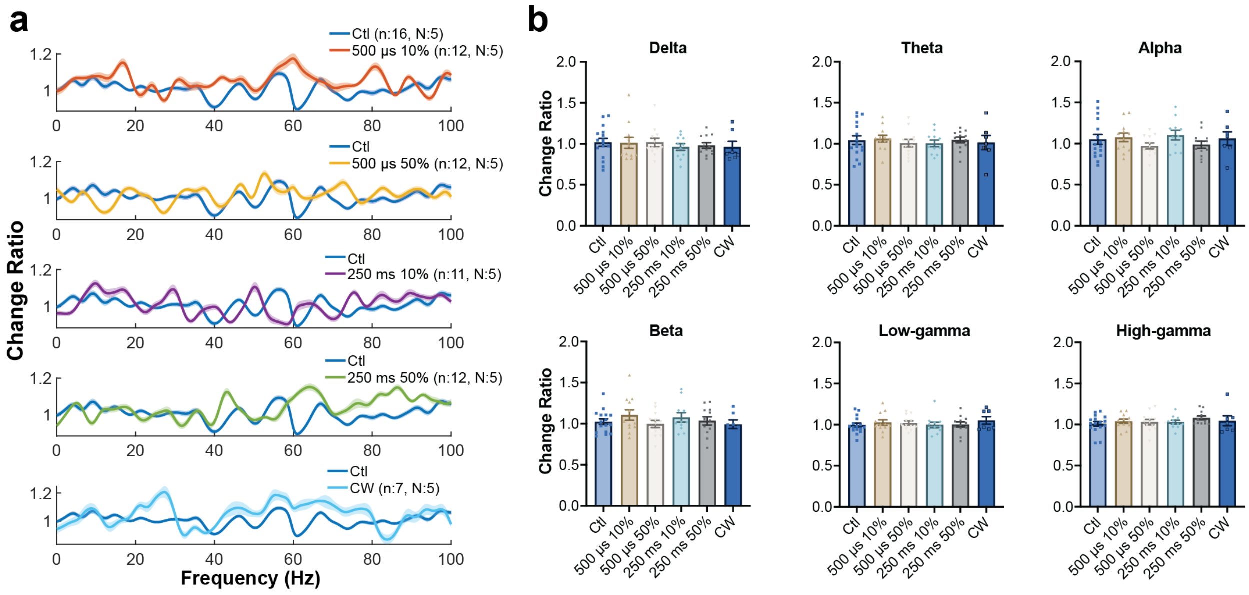

Supplementary Figure 9. Ketamine eliminates local field potential (LFP) changes induced by 40 kHz ultrasound in the somatosensory cortex. (a) Power spectral density (PSD) change ratio of the LFP during ultrasound exposure in the anesthesia with ketamine. Data expressed as Mean ± SEM (n: trial number, N: animal number). (**b**) Summary of PSD change in each frequency band in ketamine. No significant change was detected in any frequency band during ultrasound exposure in the anesthesia with ketamine. Each dot represents each trial. Data expressed as Mean ± SEM. Statistics: One-way ANOVA with Dunnett's multiple comparison test.

Supplementary Figure 10

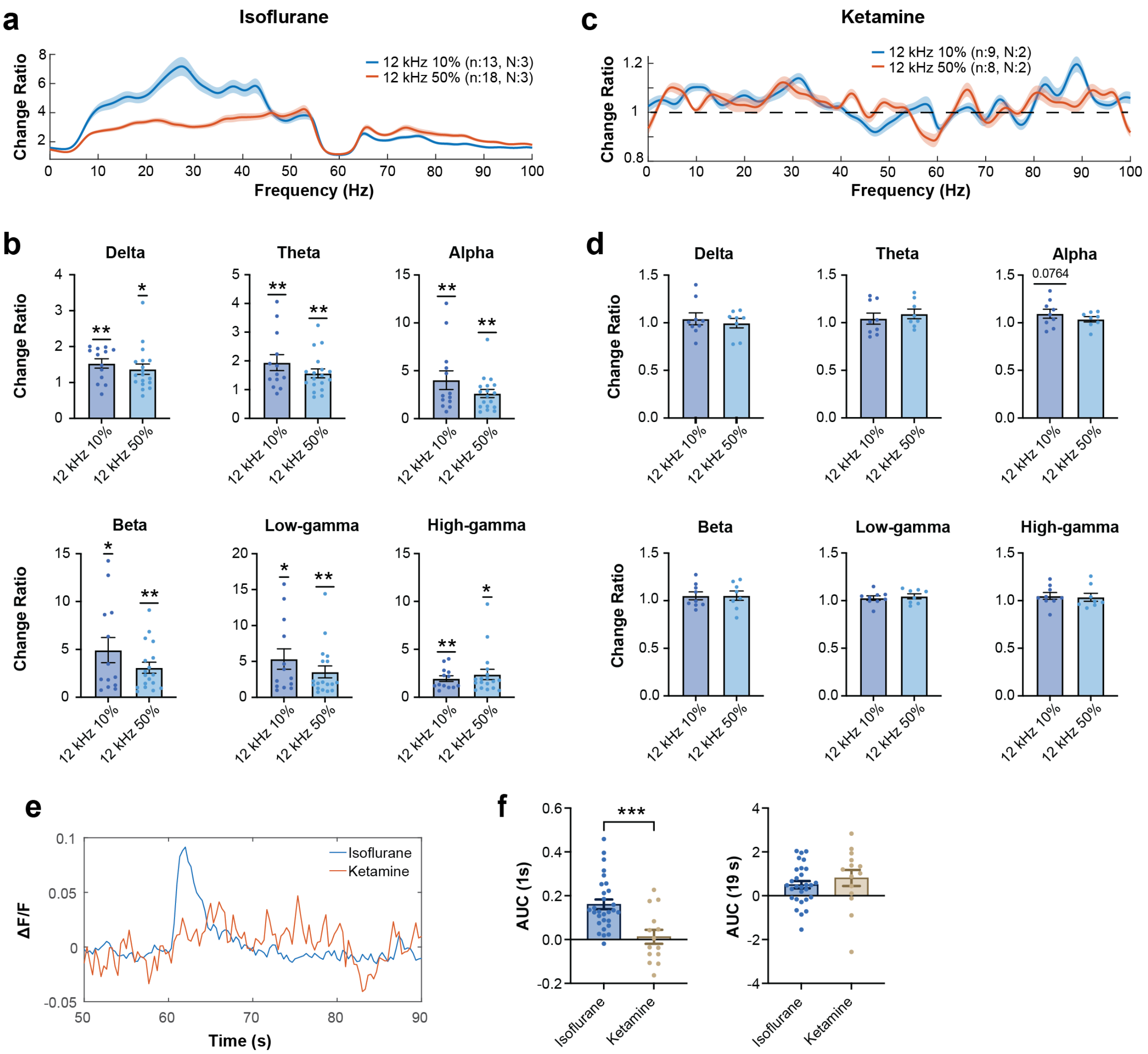

Supplementary Figure 10. Ketamine eliminates local field potential (LFP) changes induced by 12 kHz sound in the somatosensory cortex. (a) Power spectral density (PSD) change ratio of the LFP during sound exposure in the anesthesia with isoflurane. Note the increase in the overall power from 1 – 100 Hz. Dotted line indicates the change ratio at 1, suggesting no change. **(b)** Summary of PSD change in each frequency band during sound exposure under anesthesia with isoflurane. All frequency bands show significant changes when compared with theoretical no change (change ratio equals 1). **(c)** PSD change ratio of the LFP during sound exposure in the anesthesia with ketamine. No apparent change was noted. Note the different scales in A and C. (n: trial number, N: animal number). **(d)** Summary of PSD change in each frequency band during sound exposure under anesthesia with ketamine. No significant changes were detected in any frequency band when

compared with theoretical no change (change ratio equals 1). (**e**) Comparison of Ca^{2+} responses to 50% sound between under anesthesia of isoflurane and ketamine in the somatosensory cortex (SC). (**f**) Summary of the area under the curve (AUC) of the first second and during 2 to 20 second of the Ca^{2+} signal. AUCs of the first second in ketamine were significantly lower than in isoflurane. However, AUCs of the later 19 second in ketamine were not significantly different from in isoflurane. (Statistics: One sample Wilcoxon test in (b) and (d), Mann-Whitney test in (f). * $p < 0.05$, ** $p < 0.01$, $p < 0.001$). Data are expressed as Mean ± SEM, and each dot represents one trial.

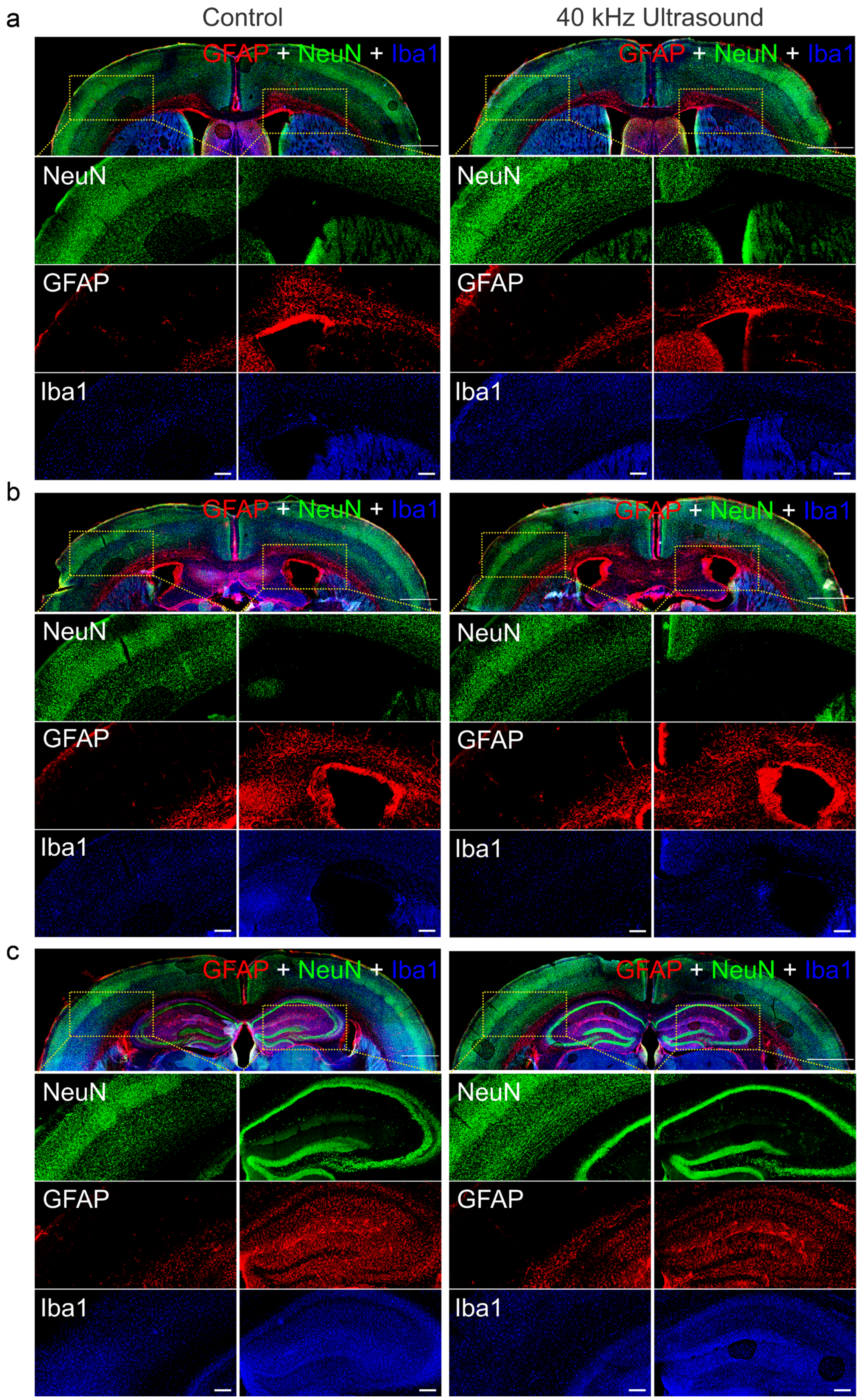
a
Control
40 kHz Ultrasound
GFAP + NeuN + Iba1
NeuN
GFAP
Iba1
b
c

Supplementary Figure 11. Histology assessment of tissues 6 months after a single 10% 500 μs 40 kHz ultrasound exposure. (a) Coronal sections at 1 mm posterior of bregma from a control (left) and a sonicated (right) mouse. White boxes indicate regions of the enlarged images. **(b)** Coronal sections at 1 mm posterior of bregma. **(c)** Coronal sections at 2 mm posterior of bregma. Some nonspecific GFAP and Iba1 reactions are noted in both control and sonicated slices. Scale bars in montage: 1 mm, scale bars in enlarged images: 200 μm.

Supplementary References

1. A. Fomenko, K. S. Chen, J. F. Nankoo, J. Saravanamuttu, Y. Wang, M. El-Baba, X. Xia, S. S. Seerala, K. Hynynen, A. M. Lozano, R. Chen, Systematic examination of low-intensity ultrasound parameters on human motor cortex excitability and behavior. *Elife* **9**, (2020).
