# Supplementary material for: Low frequency ultrasound elicits broad cortical responses inhibited by ketamine in mice
Source: Commun Eng. 2024 Aug 27;3:120. doi: 10.1038/s44172-024-00269-2 (PMC11349898; doi:10.1038/s44172-024-00269-2)
Supplement: Supplementary file 2 — Reporting Summary [file 44172_2024_269_MOESM2_ESM.pdf]

nature portfolio

Corresponding author(s): Meijun Ye

Last updated by author(s): 07/05/2024

Reporting Summary

Nature Portfolio wishes to improve the reproducibility of the work that we publish. This form provides structure for consistency and transparency in reporting. For further information on Nature Portfolio policies, see our Editorial Policies and the Editorial Policy Checklist.

Statistics

For all statistical analyses, confirm that the following items are present in the figure legend, table legend, main text, or Methods section.

n/a	Confirmed	
☐	☒	The exact sample size (*n*) for each experimental group/condition, given as a discrete number and unit of measurement
☐	☒	A statement on whether measurements were taken from distinct samples or whether the same sample was measured repeatedly
☐	☒	The statistical test(s) used AND whether they are one- or two-sided<br>*Only common tests should be described solely by name; describe more complex techniques in the Methods section.*
☐	☒	A description of all covariates tested
☐	☒	A description of any assumptions or corrections, such as tests of normality and adjustment for multiple comparisons
☐	☒	A full description of the statistical parameters including central tendency (e.g. means) or other basic estimates (e.g. regression coefficient) AND variation (e.g. standard deviation) or associated estimates of uncertainty (e.g. confidence intervals)
☐	☒	For null hypothesis testing, the test statistic (e.g. *F*, *t*, *r*) with confidence intervals, effect sizes, degrees of freedom and *P* value noted<br>*Give P values as exact values whenever suitable.*
☒	☐	For Bayesian analysis, information on the choice of priors and Markov chain Monte Carlo settings
☒	☐	For hierarchical and complex designs, identification of the appropriate level for tests and full reporting of outcomes
☒	☐	Estimates of effect sizes (e.g. Cohen's *d*, Pearson's *r*), indicating how they were calculated

Our web collection on statistics for biologists contains articles on many of the points above.

Software and code

Policy information about availability of computer code

Data collection	Animals were imaged on a Movable Objective TPM Microscope (MoM, Sutter Instrument) with MScan 2.0 software.<br>Electrophysiological signals in vivo were acquired simultaneously with TPM calcium imaging via a Scout processor, Nano2+Stim front end and Trellis software (Ripple Neuro, Salt Lake City, UT)<br>Calcium imaging in brain slices was performed with Zen 3.4 Pro software (Carl Zeiss Microscopy GmbH).
Data analysis	MATLAB 2023b was used for processing of Calcium data and Electrophysiology data.<br>All statistics were conducted with Graphpad Prism 9.0 and later upgraded to 10.0.

For manuscripts utilizing custom algorithms or software that are central to the research but not yet described in published literature, software must be made available to editors and reviewers. We strongly encourage code deposition in a community repository (e.g. GitHub). See the Nature Portfolio guidelines for submitting code & software for further information.

Data

Policy information about availability of data

All manuscripts must include a data availability statement. This statement should provide the following information, where applicable:

- Accession codes, unique identifiers, or web links for publicly available datasets
- A description of any restrictions on data availability
- For clinical datasets or third party data, please ensure that the statement adheres to our policy

All data needed to evaluate the conclusions are present in this paper and/or the Supplementary Information. All other relevant data supporting the findings of this study are available from the corresponding authors upon request.

Human research participants

Policy information about studies involving human research participants and Sex and Gender in Research.

Reporting on sex and gender	N.A.
Population characteristics	N.A.
Recruitment	N.A.
Ethics oversight	N.A.

Note that full information on the approval of the study protocol must also be provided in the manuscript.

Field-specific reporting

Please select the one below that is the best fit for your research. If you are not sure, read the appropriate sections before making your selection.

☒ Life sciences ☐ Behavioural & social sciences ☐ Ecological, evolutionary & environmental sciences

For a reference copy of the document with all sections, see nature.com/documents/nr-reporting-summary-flat.pdf

Life sciences study design

All studies must disclose on these points even when the disclosure is negative.

Sample size	*Describe how sample size was determined, detailing any statistical methods used to predetermine sample size OR if no sample-size calculation was performed, describe how sample sizes were chosen and provide a rationale for why these sample sizes are sufficient.*
Data exclusions	In vivo imaging data with the related Electrophysiological data was excluded under the following conditions: animal movement, water level low, electromagnetic interference in the imaging system, fluorescence with low signal noise ratio at certain area in the craniotomy window.
Replication	All the reported results were repeatable. Replicates were used in all experiments, as noted in the figure legends and in Methods.
Randomization	Multiple ultrasound dosages and sham control were delivered in a random order to each animal in each ROI to avoid region, anesthesia duration, and exposure sequency bias. Between each exposure, 5 mins of interval was applied. The dosage delivery order was also randomized between animals.
Blinding	During the calcium imaging analysis, the analyzers were blinded to the dosage of the exposure while selecting individual cells with attempt to include all cells in the imaging field. Group or dosage information associated with each calcium time series were blinded for calcium imaging analysis until statistical analysis stage.

Reporting for specific materials, systems and methods

We require information from authors about some types of materials, experimental systems and methods used in many studies. Here, indicate whether each material, system or method listed is relevant to your study. If you are not sure if a list item applies to your research, read the appropriate section before selecting a response.

Materials & experimental systems

n/a	Involved in the study
☐	☒ Antibodies
☒	☐ Eukaryotic cell lines
☒	☐ Palaeontology and archaeology
☐	☒ Animals and other organisms
☒	☐ Clinical data
☒	☐ Dual use research of concern

Methods

n/a	Involved in the study
☒	☐ ChIP-seq
☒	☐ Flow cytometry
☒	☐ MRI-based neuroimaging

Antibodies

Antibodies used	Primary antibodies against NeuN (pChicken; Millipore Sigma) to stain neuronal cytoplasms, GFAP (mRat; ThermoFisher, Waltham, Massachusetts) for astrocytes, and Iba1 (pRabbit; Wako, Osaka, Japan) for microglia.<br>Secondary antibodies raised in goat: Alexa Fluor 405 (anti-chicken; Abcam, Cambridge, Massachusetts), Alexa Fluor 488 (anti-rat, ThermoFisher), and Alexa Fluor 647 (anti-rabbit, ThermoFisher)
Validation	NeuN (pChicken; Millipore Sigma): Evaluated by Western Blot in mouse brain E16 tissue lysate. (Manufacture website)<br>GFAP (mRat; ThermoFisher, Waltham, Massachusetts): Western blot analysis was performed on tissue extracts of Mouse brain. (Manufacture website)<br>Iba1 (pRabbit; Wako, Osaka, Japan): Electrophoresis test. (Manufacture website)

Animals and other research organisms

Policy information about studies involving animals; ARRIVE guidelines recommended for reporting animal research, and Sex and Gender in Research

Laboratory animals	Adult male Thy1-GCaMP6s transgenic mice (C57BL/6J-Tg (Thy1-GCaMP6s)GP4.3Dkim/J, Jackson Laboratory, Bar Harbor, Maine, Stock No: 024275) aged 2 to 6 months were used for calcium imaging.
Wild animals	No wild animal was used for calcium imaging.
Reporting on sex	No previous literature has reported sex-specific responses for ultrasound induced calcium activities in mice to the best of our knowledge. We acknowledging the limitations of using only male animals and future studies include both sexes would be meaningful.
Field-collected samples	The study did not involve samples collected from the field.
Ethics oversight	All animal use procedures in this study were approved by the Food and Drug Administration (FDA) White Oak Institutional Animal Care and Use Committee and Animal Care and Use Review Office (ACURO) of the Department of Defense. All procedures complied with the National Institutes of Health Guide for the Care and Use of Laboratory Animals.

Note that full information on the approval of the study protocol must also be provided in the manuscript.
